# Identifying and avoiding design related biases in observational studies using the target trial framework

**DOI:** 10.1136/bmjmed-2024-001280

**Published:** 2026-02-20

**Authors:** Harrison J Hansford, Nazrul Islam, Hopin Lee, Barbra A Dickerman, Aidan G Cashin

**Affiliations:** 1 School of Health Sciences, Faculty of Medicine and Health, UNSW Sydney, Sydney, NSW, Australia; 2 Centre for Pain IMPACT, Neuroscience Research Australia, Randwick, NSW, Australia; 3 Primary Care Research Centre, Faculty of Medicine, University of Southampton, Southampton, UK; 4 University of Exeter Medical School, Exeter, UK; 5 EMEA Methods & Evidence Generation, IQVIA, London, UK; 6 CAUSALab, Department of Epidemiology, Harvard T H Chan School of Public Health, Boston, MA, USA; 7 School of Population Health, Faculty of Medicine and Health, UNSW Sydney, Sydney, NSW, Australia

**Keywords:** Epidemiology, Medicine, Public health, Research design

## Abstract

Observational studies are necessary to provide evidence to inform decision making in the absence of a relevant randomised trial. Although commonly criticised for potential problems due to confounding bias, design related biases in observational studies are often overlooked yet highly prevalent. Design related biases occur because of decisions made by researchers during analyses of observational data. Common design related biases include bias related to selection and treatment misclassification, resulting from misalignment of eligibility ascertainment, treatment strategy assignment, and start of follow-up. Conceptualising the analysis of observational data to estimate the causal effects of interventions as an attempt to explicitly emulate a target trial can help avoid design related biases, so that investigators can instead focus on data related biases (eg, confounding, measurement error) not directly addressed by the framework. Target trial emulation may also help readers appraise an observational study when transparently reported. This article aims to help readers of observational studies identify and avoid design related biases to support the use of observational evidence to inform clinical and policy decision making.

**Key messages**

- Design related biases are commonly introduced by investigators in observational studies of interventions
- Prevalent user bias and inclusion of immortal time are common and important design related biases in observational studies of interventions
- The target trial framework can help investigators identify and avoid design related biases in observational studies of interventions

## Introduction

 Randomised trials are the preferred approach to estimate the causal effects of medical interventions.^1 2^ However, it is often not feasible to conduct randomised trials to investigate the long term safety and effectiveness of interventions,^3^ identify specific groups who benefit (or are harmed) most from interventions,^4^ or to provide evidence when timely information is needed for decision making.^5^ Routinely collected, observational data (eg, in the form of electronic health records and claims data) have become increasingly available for research^6^ and are frequently used to inform decision making where randomised trials are not available.^7 8^ Because of the ability to complement evidence from randomised trials, regulators such as the European Medicines Agency are reviewing the use of observational data to inform regulatory decision making around the safety and effectiveness of medicines.^9^ Despite the necessity of using observational data to inform health decision making in the absence of a relevant randomised trial, clear challenges exist in drawing valid causal inferences from observational data.^10^

Clinicians and policymakers are often hesitant to rely on evidence from observational studies, because of concerns of confounding bias due to the lack of randomisation.^10 11^ However, design related biases^12^ can explain discrepancies between findings in observational data and randomised trials, sometimes even more so than confounding.^13^,^17^ These biases are introduced when observational studies are designed in a manner that deviates from the design principles of a randomised trial.^18^ For example, well designed randomised trials will follow participants from the time of randomisation (treatment strategy assignment) immediately after meeting eligibility criteria, such that there is a clear “time zero.” In observational studies of longitudinal data, the timing of eligibility, treatment strategy assignment, and start of follow-up are not naturally aligned, and analysts must therefore define these timepoints retrospectively.^19^ The choice of these timepoints is where errors commonly occur.^13 20^ In a cross sectional study of 200 observational studies of drug interventions,^21^ Yaacoub et al highlighted that over 75% of studies were subject to at least one avoidable design related bias caused by the misalignment of time zero.

All investigators and readers—including clinicians, peer reviewers, and other health and policy decision makers—must be able to identify and avoid these often-overlooked biases related to design, to maximise the usefulness of observational data for decision making.^22 23^ This article aims to provide a detailed outline of how design related biases may occur and how target trial emulation can help avoid these biases, with reference to the growing literature on this topic in health and medical research.^1213 17 19 24^,^29 29^ Although target trial emulation can help to avoid design related biases, it does not eliminate the potential for biases inherent to the observational data, such as confounding.^25^ While not the focus of this article, we briefly discuss approaches to handle confounding.^36^ We also highlight the importance of transparent reporting for observational studies of interventions to be appraised and used in practice.^37^ Key terms used in this article are defined in table 1, with references to articles with more detail.

**Table 1:** Key terms that relate to design related biases and target trial emulation

Term	Definition
Target trial	Hypothetical randomised trial that would be conducted to answer the question of interest, with respect to the variables available in the observational data.^25^
Target trial emulation	Process of mapping the analysis of observational data to the components of the target trial (eligibility criteria, treatment strategies, assignment procedures, follow-up, outcomes, causal contrast, data analysis plan) with specification of the assumptions necessary for causal inference.^25^
Time zero	Time at which follow-up begins, which should align with the time of eligibility ascertainment and treatment strategy assignment.^12^
Design related bias	Biases introduced by investigator decisions about the design of an observational study, often occurring when investigators deviate from the design principles of a randomised trial.^13^
Selection bias	Systematic inclusion or exclusion of individuals into an analysis resulting in a biased treatment effect estimate.^19^
Misclassification of treatment assignment	Errors when classifying individuals into a treatment strategy caused by the use of information that emerges after the beginning of follow-up to assign individuals to a treatment strategy. This scenario often occurs when baseline information alone is insufficient to classify individuals into a treatment strategy.^19^
Clone-censor-weight method	Analytical approach where the study dataset is replicated (cloned), and replicates (clones) are assigned to each treatment strategy with compatible data at baseline and are censored when they deviate from their assigned strategy; inverse probability weights are then applied to overcome biases introduced by the censoring. This approach is used to handle scenarios in which baseline information alone is insufficient to classify individuals into a treatment strategy.^32 34^
Sequential trial emulation	Analytical approach where a series of hypothetical target trials are conceptualised and emulated where individuals who are eligible at multiple times are included at each time of eligibility. This approach is used to handle scenarios where individuals may meet the eligibility criteria at multiple times over follow-up and is more statistically efficient than selecting only one of those times as time zero.^53^

## Identifying common design related biases in observational studies

The design related biases described in this section all stem from deviation from the design principles of a randomised trial. These biases include selection bias due to the inclusion of prevalent users, and immortal time generated when information after the start of follow-up is used to ascertain eligibility or classify individuals into treatment strategies (discussed further below). While desirable, predicting the magnitude and direction of these design related biases is challenging.^38^ We hope that by providing the structure of how these biases occur in this article, readers may be better placed to assess the potential direction of bias in the context of their own research questions or studies they may appraise.

### Prevalent user bias

**Figure 1:**
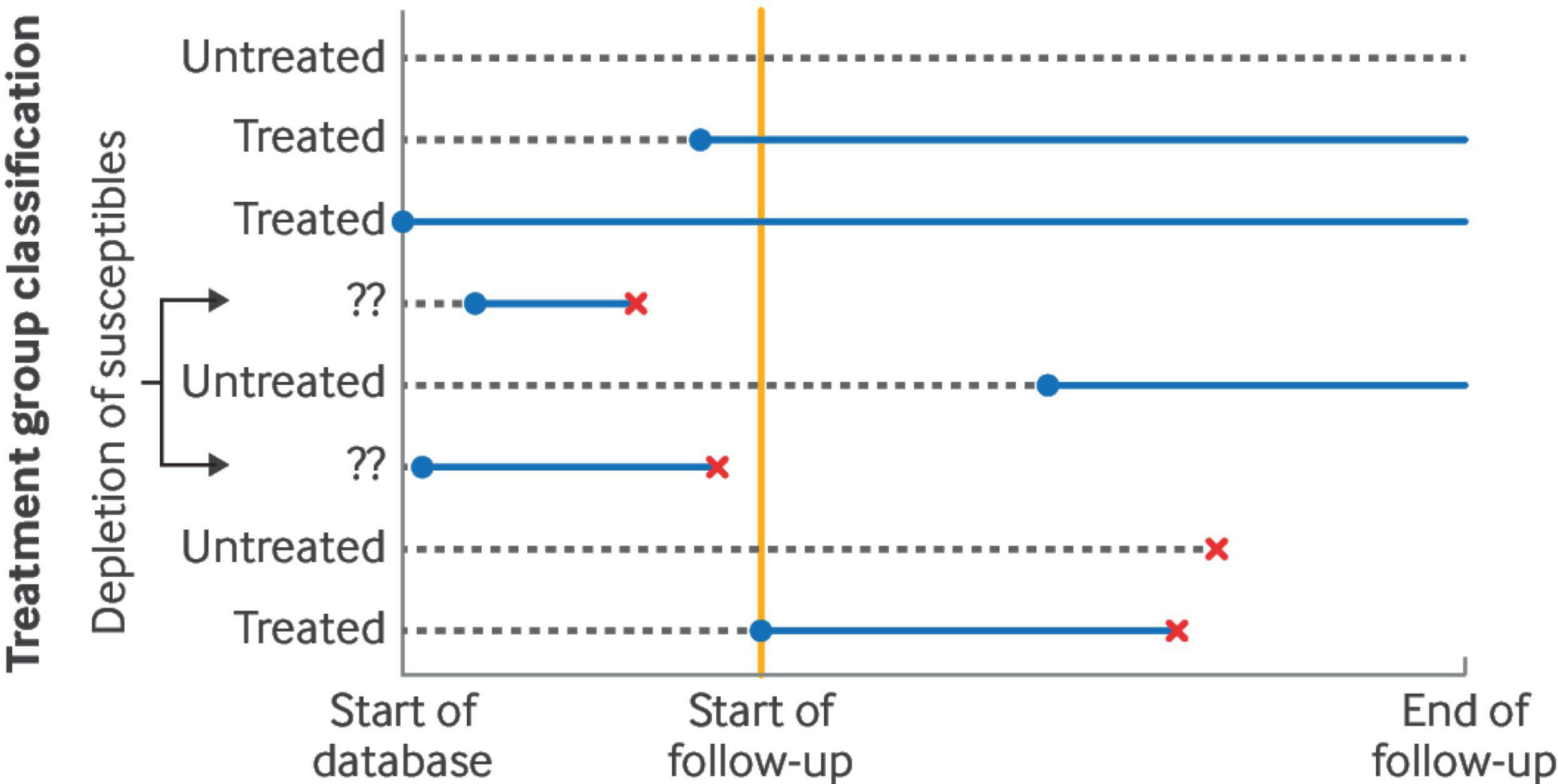
Graphical depiction of prevalent user bias. Blue lines=treatment; dashed lines=no treatment; red crosses=people who died, experienced the outcome of interest, or were lost to follow-up; yellow line=start of follow-up (ie, time zero). Depletion of susceptibles occurs when individuals who experience outcomes during treatment before the start of follow-up are systematically excluded. Figure adapted from Fu et al^24^

Studies that include prevalent users of treatment are prone to selection bias (sometimes referred to as prevalent user bias, or depletion of susceptibles).^39 40^ This bias arises when the treated group includes individuals who had been taking treatment for some time before the start of follow-up and who remained alive and event-free until the start of follow-up (figure 1).

### Immortal time

Immortal time refers to a period of time included in the analysis where individuals cannot experience the outcome of interest owing to choices about the study design.^12 28 41^ In a randomised trial, all outcomes that occur after eligibility ascertainment, treatment strategy assignment, and the start of follow-up are attributed to the appropriate treatment strategy. However, in observational analyses, when information that is ascertained after the start of follow-up is used to inform eligibility (or classification into treatment strategies), immortal time may be introduced. Below are examples of specific design decisions that can introduce immortal time.

#### Use of information emerging after treatment assignment to define eligibility (selection bias)

**Figure 2:**
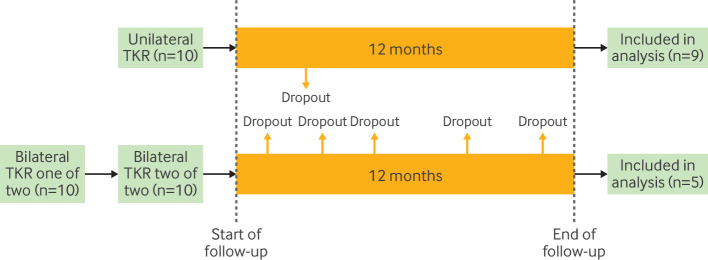
Immortal time due to selection bias. Immortal time can arise when eligibility criteria (eg, 12 month follow-up response) are applied after treatment assignment (eg, unilateral *v* bilateral total knee replacement (TKR)) and the start of follow-up, thereby creating a period of immortal time between baseline and eligibility ascertainment where the outcome cannot occur for included individuals (ie, everyone who is included in the analysis must have survived and not dropped out of the study, and is therefore “immortal”). This example also includes bias due to misclassification of treatment assignment (see Errors in classifying individuals into treatment strategies, below)

Using information that emerges after treatment assignment (eg, adherence to treatment strategy, duration of treatment use, providing follow-up data) to define the eligibility criteria induces selection bias when the analysis starts the follow-up at treatment assignment. In a study comparing the effect of unilateral total knee replacement with staged bilateral total knee replacement for the outcome of functional impairment, investigators included only participants who provided follow-up data at 12 months after surgery.^29^ This decision will result in a biased treatment effect estimate if response rates differ on the basis of the intervention, and if there are common causes of response and the outcome of interest (eg, persistent pain). In general, if information measured after treatment strategy assignment and the start of follow-up is used to determine eligibility, the treatment differentially influences those eligibility criteria, and there are common causes of the eligibility criteria and the outcome, then the treatment effect can be biased (figure 2).^21^ In a well designed and executed randomised trial, the time of eligibility ascertainment is anchored to the time of treatment strategy assignment (randomisation) and the start of follow-up, avoiding this issue.

#### Errors in classifying individuals into treatment strategies (misclassification bias)

**Figure 3:**
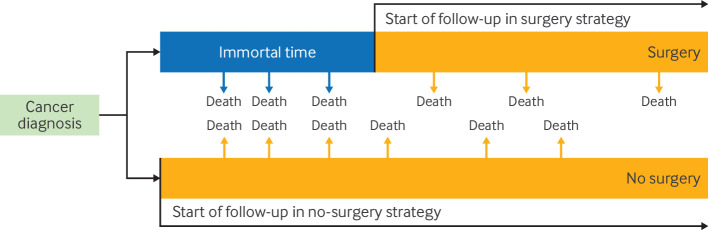
Immortal time created due to misclassification of treatment assignment. This scenario can arise when treatment strategies (eg, surgery within six months or no surgery) are assigned using information after the start of follow-up (eg, cancer diagnosis), which can result in events being misattributed to one of the treatment groups (eg, early deaths marked by blue arrows in the figure are misattributed to the strategy of no surgery)

Immortal time can also arise when individuals are classified into a treatment strategy that differs from the one they should have been assigned to. This misclassification often occurs when treatment strategies under comparison cannot be distinguished at time zero. In a study that compared surgery with no surgery for lung cancer in elderly people, a grace period of six months was necessary to allow individuals time from their diagnosis to receive surgery, because surgery often does not occur immediately after diagnosis.^30^ Using only information at cancer diagnosis, it is unclear which treatment strategy individuals should be classified to, because their data at baseline may be compatible with both strategies (ie, surgery within six months or no surgery). If investigators look forward and classify individuals who undergo surgery within six months to the surgery strategy, all individuals in that strategy would have survived until their surgery. Therefore, in this scenario, events (eg, death) that occur before surgery will be attributed to the strategy of no surgery. This scenario may result in up to six months of immortal time for participants classified to the surgery strategy (figure 3).^30^ In a randomised trial, participants would be allocated prospectively to either strategy and events attributed to the strategy assigned at baseline, avoiding this issue. To avoid introducing immortal time, analysts of observational data can use approaches that overcome the lack of knowledge on what treatment strategy individuals were intended to be allocated, without using information that is measured after baseline.

## Avoiding design related biases with target trial emulation

Carefully mapping the analysis of an observational study to the framework of a randomised trial that would answer the question of interest, known as target trial emulation, can help investigators avoid design related biases.^25 38 42^ The emulation of a target trial involves two steps. The first step is to design the hypothetical pragmatic trial—the target trial—that would answer the question of interest, and use only variables that are available in the observational data source (ie, the target trial is able to be emulated with the available data).^25^ The target trial can be considered a detailed description of the causal question (or estimand) that is being targeted.^25^ The critical components to be considered when specifying the target trial are: eligibility criteria, treatment strategies, treatment assignment, outcomes, follow-up, and causal contrasts of interest (eg, intention-to-treat effect), as well as any identifying assumptions and the data analysis plan.^14 25^ After the target trial is specified, the second step is to map the components of the target trial to the observational data (ie, emulate the target trial) and apply appropriate analytical techniques to adjust for confounding.^27 43 44^ There may be instances where data on all variables needed to emulate the target trial are not available; however, still specifying the target trial can help readers understand these limitations.^45^

Several approaches under the target trial framework can help investigators avoid inducing design biases common in observational studies, such as to avoid inclusion of individuals already taking treatment at baseline (avoiding prevalent user bias) and to avoid the use of information that is available after baseline to assign individuals to treatment strategies or to define eligibility (avoiding immortal time).

### Avoiding prevalent user bias

Prevalent user bias can be avoided by applying eligibility criteria in the target trial that restrict entry into the hypothetical trial to individuals who have not used the treatment for a defined period. This same criterion must be applied in the analysis of the observational data (emulation) and is often implemented using a so-called look-back period where individuals cannot have used the treatment for some defined period before baseline (time zero). Including only individuals who are just starting treatment has also been termed a new user design or incident user design in pharmacoepidemiology.^46^ There are also occasions where prevalent users are of interest, and designs are emerging to handle the depletion of susceptibles appropriately^47 48^; however, such designs can be challenging to apply and interpret.^49^

An example of prevalent user bias occurred in observational studies investigating hormone replacement therapy for the prevention of coronary heart disease.^50 51^ Grodstein et al^51^ compared women using hormone therapy (prevalent users) to women who did not use hormone therapy. The observational study suggested a reduced risk of coronary heart disease among users of hormone therapy compared with non-users,^51^ a result that conflicted with a later randomised trial showing an increased risk of coronary heart disease for those assigned to initiate hormone therapy compared with placebo.^52^ The same data in Grodstein et al^51^ were used by Hernán et al,^50^ although these investigators adhered more closely to the principles of the randomised trial by comparing initiators to non-initiators and found a similarly increased risk of coronary heart disease to the trial.^50^ In the original observational study,^51^ the inclusion of prevalent users introduced bias as individuals who were susceptible to adverse events of hormone therapy or who had coronary heart disease were less likely to be included in the study (depletion of susceptibles), resulting in an underestimation of the true risk of hormone therapy. The comparison between initiators and non-initiators of treatment better aligns with common clinical decisions about whether to start a new treatment, and how a randomised trial would be conducted.^50^

### Avoiding immortal time

Immortal time is introduced by the selection of individuals based on eligibility criteria defined after treatment strategy assignment, when the analysis starts the follow-up at assignment; or the misclassification of individuals into treatment strategies based on information available after eligibility ascertainment and the start of follow-up. These biases do not exist in well designed randomised trials, which have negligible gaps between eligibility ascertainment, treatment strategy assignment, and the start of follow-up. Specifying a target trial that similarly aligns these features will avoid the introduction of immortal time.^12 53^

Immortal time can have substantial impacts on study results. To illustrate this difference, Kuehne et al systematically applied different approaches to estimate the effect of LOT2 (a type of chemotherapy for ovarian cancer), including designs subject to immortal time, to highlight how the estimates change with design decisions.^17^ With the naive approach subject to immortal time, the researchers estimated a hazard ratio of 0.56 (95% confidence interval (CI) 0.49 to 0.64). By contrast, when they emulated the design of an index trial, they estimated a hazard ratio of 1.12 (0.96 to 1.28), which was compatible with the results of the trial.^17^

This alignment is straightforward when estimating the effect of receiving an intervention that occurs at a single timepoint such as a surgery or single dose vaccine. However, this alignment is more complex when estimating the effect of treatment strategies that are indistinguishable at baseline, such as when the effect of the timing of treatment is of interest (eg, start treatment within six months of diagnosis or after six months). Several strategies have been developed to handle these situations.^32^

#### Clone-censor-weight approach

An approach to handle questions where treatment strategies are indistinguishable at the start of follow-up, in conjunction with the target trial framework, is the clone-censor-weight approach.^32 34 54^ To implement this, investigators can clone participants—that is, replicate individuals, and assign replicates to as many treatment strategies as their data are compatible with at baseline. Because a person's observed treatment pattern becomes incompatible with their assigned strategy, their follow-up in that treatment strategy is artificially stopped or censored by the investigator at the time of deviation from their assigned strategy. This artificial censoring ensures that individuals classified into each strategy are only included in follow-up while they adhere to their assigned treatment strategy. However, if adherence is differential (non-random) between the treatment strategies, the artificial censoring may induce selection bias,^55^ requiring a third step: weighting. Inverse probability weights are estimated and applied to adjust for the selection bias induced by this censoring.^32 34^ For further discussion, Hernán and Gaber et al provide in-depth explanations of how to conduct a clone-censor-weight analysis.^32 34^

**Figure 4:**
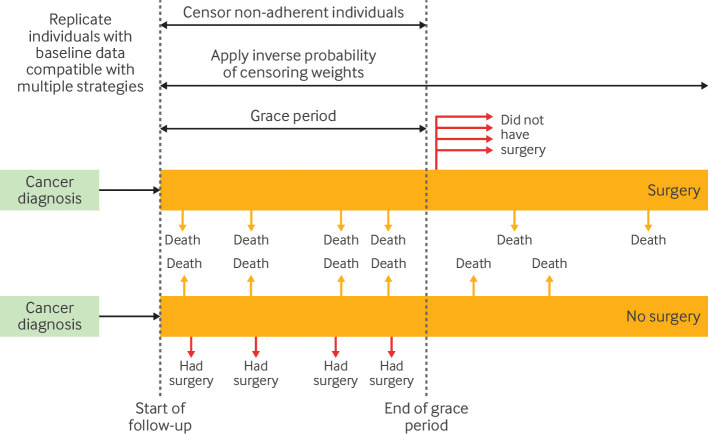
Illustration of clone-censor-weight approach. Red arrows=deviation from treatment strategies leading to censoring (eg, when individuals classified to receive no surgery, end up receiving surgery, or when individuals classified to surgery within a certain grace period strategy do not receive surgery by the end of the grace period)

An example of where the clone-censor-weight approach may be helpful is when comparing treatment strategies that involve a grace period for initiating treatment (ie, a predefined period of time after baseline during which treatment can begin, reflecting delays common in clinical practice) (figure 4).^34^ Consider the example of comparing receipt of surgery within six months (the grace period) of baseline (lung cancer diagnosis) with no surgery over follow-up (figure 3). Since it is unclear which treatment strategy individuals should be assigned to at baseline, all individuals who have baseline data compatible with both treatment strategies (eg, have not had surgery immediately on cancer diagnosis) are replicated. Then, as replicates classified at baseline to no surgery then have surgery during follow-up, they are censored at that time because their data are no longer compatible with their strategy assigned at baseline. Similarly, for replicates classified to the surgery strategy who do not have surgery by the end of the grace period, they are censored at six months. Inverse probability weights are applied throughout the follow-up period to account for the censoring.

Applying target trial emulation with cloning, censoring, and weighting prevented immortal time in a recent example by Boyne et al investigating survival after short or longer duration of adjuvant chemotherapy for colon cancer.^56^ Before this study, findings from a randomised trial (the IDEA trial)^57^ conflicted with those from observational studies. In these previous observational studies, information emerging after baseline (achieved duration of treatment) was used to classify individuals into treatment strategies, leading to immortal time.^56^ It demonstrated that with the naive approach taken in previous observational studies, estimates suggested that shorter durations of treatment substantially worsened survival (hazard ratio 3.33 (95% CI 1.04 to 10.65)).^56^ However, when information emerging after baseline was not used to classify individuals into treatment strategies, and cloning, censoring, and weighting was applied instead, the estimates were compatible with those from the IDEA trial (emulation hazard ratio 0.96 (95% CI 0.43 to 2.14); trial hazard ratio 0.96 (95% CI 0.85 to 1.08)).^56^ Aside from the clone-censor-weight approach, we note that the plug-in g formula is another approach available to analysts; however, it is not described in this article with further reading available elsewhere.^58 59^

#### Redefining the treatment strategies

Another approach to avoid immortal time where treatment strategies are not distinguishable at baseline is reformulating the causal question to make the strategies distinguishable at baseline.^33 54^ Consider the example outlined previously: comparing receipt of surgery within six months of baseline with no surgery over follow-up. Investigators could reformulate the first strategy as receipt of surgery at baseline—that is, without a grace period. Therefore, individuals who initiate surgery at baseline will be classified into the surgery strategy, and into the no-surgery strategy otherwise. This approach avoids the introduction of immortal time due to treatment strategy misclassification; however, whether the new causal question (eg, without grace periods, which may be realistic in many real world treatment strategies) is still of interest should be considered.

**Figure 5:**
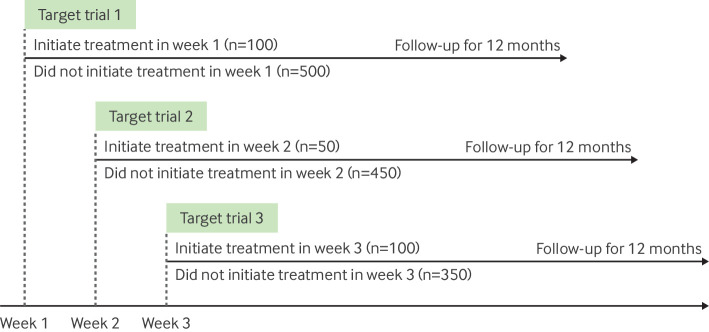
Emulation of sequential trials. Each target trial recruits each week (or other relevant time period, where follow-up begins (time zero)), where investigators classify individuals into the treatment strategy their data is compatible with during that week, and then follows individuals for 12 months

In principle, using only one eligible time (eg, the first, or a random eligible time) would be an unbiased approach, because it relies only on baseline information to classify individuals into a treatment strategy.^12 19^ However, a large amount of information could be lost, leading to less precise estimates.^12^ A more efficient approach could be to instead emulate a sequence of nested trials, each initiated at a certain calendar period (eg, each day, week, or month), which becomes time zero of a given nested trial.^33^ For example, eligible individuals who initiate surgery in the first week of each nested trial could be compared with eligible individuals who do not have surgery in that week (figure 5). Information across these trials can then be pooled to provide a single estimate of the treatment effect.

Applying target trial emulation with sequential trial emulation prevented design related biases in an evaluation of statins and risk of cancer.^13^ In earlier observational analyses, investigators reported a substantially lower risk of cancer among statin users than among non-users (odds ratio for lung cancer of 0.23 (95% CI 0.20 to 0.26), comparing long term (>4 years) statin users with non-users).^60^ These analyses had two flaws previously discussed^31 50^: post-baseline information on achieved duration of statin use was used to classify individuals into treatment strategies, generating immortal time, and prevalent users of treatment were included, leading to selection bias. Dickerman et al reframed this analysis as a target trial with sequential trial emulation and classified individuals into one of two strategies (statin initiators *v* non-initiators) based on information available at the baseline of each sequential target trial, finding a null effect (hazard ratio 1.02 (95% CI 0.99 to 1.05)), consistent with meta-analyses of randomised trials.^61 62^ Despite the potential benefits of sequential trial emulation, the approach may become computationally intensive in large datasets.^63^

## Common approaches to handle confounding

Although target trial emulation can help to avoid the design related biases outlined above, it does not eliminate data related biases, such as confounding or measurement error.^44^ Such biases must be dealt with through careful measurement and adjustment for key variables via appropriate analytical techniques in combination with unverifiable assumptions.^64^ Analytical approaches to adjust for confounding may involve modelling the probability of treatment (eg, inverse probability of treatment weighting^65^), the probability of the outcome (eg, multivariable outcome regression,^66^ the g formula^58 59^), or both (eg, doubly robust approaches such as targeted maximum likelihood estimation^67^ and augmented inverse probability weighting^68^). Matching, often on the propensity score,^69 70^ ^71^ may be used to adjust for time-fixed confounding for point interventions but not for time-varying confounding for sustained intervention strategies.^72 73^ Confounding by indication may be more of a concern when comparing an active intervention (eg, a drug) to no intervention; the use of an active comparator (eg, another drug) may reduce the magnitude of confounding by indication.^74^ Similarly, as with all analyses aiming to estimate causal effects of sustained intervention strategies, time-varying confounding should be managed by appropriate methods (such as inverse probability weighting^73^) when necessary.

## Transparent reporting of observational studies of interventions

Observational studies of interventions should be considered as attempts to emulate a target trial,^25 42^ and the design related issues described in this article are easier to assess when authors explicitly report their analysis as a target trial emulation.^21^ The TARGET guideline^37 75 76^ aims to assist investigators to report relevant information and support readers to identify key aspects of the target trial emulation. TARGET was not designed to be, and should not be, used as a tool to assess risk of bias or quality of a study. However, although not the intention, the TARGET guideline may help improve study conduct by serving as an educational tool and clarifying key methodological issues to be resolved by investigators. Study design diagrams, such as those described by Schneeweiss et al,^77^ can also assist investigators to communicate the timing of different aspects of the study design.^77^

## Conclusion

In settings where trials are unavailable or infeasible, observational studies may provide evidence to answer the relevant clinical question. Design related biases are common in observational studies on the comparative benefits and harms of interventions, but explicit emulation of a target trial can help investigators avoid these biases and enable readers to identify them when present. The use of the target trial framework and transparent reporting of these studies may support sound policy and clinical decisions from observational studies.

## References

[1] Cochrane AL (1972). Effectiveness and efficiency: random reflections on health services.

[2] Concato J, Shah N, Horwitz RI (2000). Randomized, controlled trials, observational studies, and the hierarchy of research designs. N Engl J Med.

[3] Matthews AA, Dahabreh IJ, Fröbert O (2022). Benchmarking Observational Analyses Before Using Them to Address Questions Trials Do Not Answer: An Application to Coronary Thrombus Aspiration. Am J Epidemiol.

[4] Moler-Zapata S, Hutchings A, O’Neill S (2023). Emulating Target Trials With Real-World Data to Inform Health Technology Assessment: Findings and Lessons From an Application to Emergency Surgery. Value Health.

[5] Hulme WJ, Williamson EJ, Green ACA (2022). Comparative effectiveness of ChAdOx1 versus BNT162b2 covid-19 vaccines in health and social care workers in England: cohort study using OpenSAFELY. BMJ.

[6] Burns L, Roux NL, Kalesnik-Orszulak R (2022). Real-World Evidence for Regulatory Decision-Making: Guidance From Around the World. Clin Ther.

[7] Hansford HJ, Cashin AG, Jones MD (2023). Reporting of Observational Studies Explicitly Aiming to Emulate Randomized Trials: A Systematic Review. JAMA Netw Open.

[8] Benchimol EI, Smeeth L, Guttmann A (2015). The REporting of studies Conducted using Observational Routinely-collected health Data (RECORD) statement. PLoS Med.

[9] European Medicines Agency (2024). Real-world evidence framework to support EU regulatory decision-making.

[10] Collins R, Bowman L, Landray M (2020). The Magic of Randomization versus the Myth of Real-World Evidence. *N Engl J Med*.

[11] Califf RM, Hernandez AF, Landray M (2020). Weighing the Benefits and Risks of Proliferating Observational Treatment Assessments: Observational Cacophony, Randomized Harmony. JAMA.

[12] Hernán MA, Sauer BC, Hernández-Díaz S (2016). Specifying a target trial prevents immortal time bias and other self-inflicted injuries in observational analyses. J Clin Epidemiol.

[13] Dickerman BA, García-Albéniz X, Logan RW (2019). Avoidable flaws in observational analyses: an application to statins and cancer. Nat Med.

[14] Hernán MA (2021). Methods of Public Health Research - Strengthening Causal Inference from Observational Data. N Engl J Med.

[15] Wang SV, Schneeweiss S, RCT-DUPLICATE Initiative (2023). Emulation of Randomized Clinical Trials With Nonrandomized Database Analyses: Results of 32 Clinical Trials. JAMA.

[16] Heyard R, Held L, Schneeweiss S (2024). Design differences and variation in results between randomised trials and non-randomised emulations: meta-analysis of RCT-DUPLICATE data. *BMJ Med*.

[17] Kuehne F, Arvandi M, Hess LM (2022). Causal analyses with target trial emulation for real-world evidence removed large self-inflicted biases: systematic bias assessment of ovarian cancer treatment effectiveness. J Clin Epidemiol.

[18] Hernán MA, Wang W, Leaf DE (2022). Target Trial Emulation: A Framework for Causal Inference From Observational Data. JAMA.

[19] Hernán MA, Sterne JAC, Higgins JPT (2025). A Structural Description of Biases That Generate Immortal Time. Epidemiology.

[20] McIntyre M, Yaacoub S, Perrodeau E (2025). Review of methods to deal with the misalignment of times of eligibility, start of follow-up, and treatment assignment in studies explicitly aimed at emulating target trials. J Clin Epidemiol.

[21] Yaacoub S, Porcher R, Pellat A (2024). Characteristics of non-randomised studies of drug treatments: cross sectional study. *BMJ Med*.

[22] Hansford HJ, McAuley JH, Cashin AG (2025). Improving reporting of observational studies of interventions: The TARGET guideline. PLoS Med.

[23] Lumbard H, PLOS Medicine Staff Editors (2025). Raising the bar for causal inference: PLOS Medicine adopts the TARGET guidelines for target trial emulation studies. PLoS Med.

[24] Fu EL, van Diepen M, Xu Y (2021). Pharmacoepidemiology for nephrologists (part 2): potential biases and how to overcome them. Clin Kidney J.

[25] Hernán MA, Dahabreh IJ, Dickerman BA (2025). The Target Trial Framework for Causal Inference From Observational Data: Why and When Is It Helpful?. *Ann Intern Med*.

[26] Fu EL (2023). Target Trial Emulation to Improve Causal Inference from Observational Data: What, Why, and How?. J Am Soc Nephrol.

[27] Desai RJ, Wang SV, Sreedhara SK (2024). Process guide for inferential studies using healthcare data from routine clinical practice to evaluate causal effects of drugs (PRINCIPLED): considerations from the FDA Sentinel Innovation Center. BMJ.

[28] Lévesque LE, Hanley JA, Kezouh A (2010). Problem of immortal time bias in cohort studies: example using statins for preventing progression of diabetes. BMJ.

[29] Larson DR, Crowson CS, Devick KL (2021). Immortal Time Bias in the Analysis of Time-to-Event Data in Orthopedics. J Arthroplasty.

[30] Maringe C, Benitez Majano S, Exarchakou A (2020). Reflection on modern methods: trial emulation in the presence of immortal-time bias. Assessing the benefit of major surgery for elderly lung cancer patients using observational data. Int J Epidemiol.

[31] Suissa S (2008). Immortal time bias in pharmaco-epidemiology. Am J Epidemiol.

[32] Hernán MA (2018). How to estimate the effect of treatment duration on survival outcomes using observational data. BMJ.

[33] García-Albéniz X, Hsu J, Hernán MA (2017). The value of explicitly emulating a target trial when using real world evidence: an application to colorectal cancer screening. Eur J Epidemiol.

[34] Gaber CE, Ghazarian AA, Strassle PD (2024). De-Mystifying the Clone-Censor-Weight Method for Causal Research Using Observational Data: A Primer for Cancer Researchers. Cancer Med.

[35] Gaber CE, Hanson KA, Kim S (2024). The Clone-Censor-Weight Method in Pharmacoepidemiologic Research: Foundations and Methodological Implementation. Curr Epidemiol Rep.

[36] Desai RJ, Franklin JM (2019). Alternative approaches for confounding adjustment in observational studies using weighting based on the propensity score: a primer for practitioners. BMJ.

[37] Cashin AG, Hansford HJ, Hernán MA (2025). Transparent reporting of observational studies emulating a target trial: the TARGET Statement. BMJ.

[38] Sterne JA, Hernán MA, Reeves BC (2016). ROBINS-I: a tool for assessing risk of bias in non-randomised studies of interventions. BMJ.

[39] Sterne JA, Hernán MA, McAleenan A (2019). Cochrane handbook for systematic reviews of interventions.

[40] Sackett DL (1979). Bias in analytic research. J Chronic Dis.

[41] Lee H, Nunan D (2020). Immortal time bias. https://catalogofbias.org/biases/immortal-time-bias/.

[42] Hernán MA (2011). With great data comes great responsibility: publishing comparative effectiveness research in epidemiology. Epidemiology (Sunnyvale).

[43] Matthews AA, Danaei G, Islam N (2022). Target trial emulation: applying principles of randomised trials to observational studies. BMJ.

[44] Hernán MA, Robins JM (2016). Using Big Data to Emulate a Target Trial When a Randomized Trial Is Not Available. Am J Epidemiol.

[45] Swanson SA (2023). The Causal Effects of Causal Inference Pedagogy. Epidemiology.

[46] Ray WA (2003). Evaluating medication effects outside of clinical trials: new-user designs. Am J Epidemiol.

[47] Suissa S, Dell’Aniello S, Renoux C (2023). The Prevalent New-user Design for Studies With no Active Comparator: The Example of Statins and Cancer. Epidemiology.

[48] Suissa S, Moodie EEM, Dell’Aniello S (2017). Prevalent new-user cohort designs for comparative drug effect studies by time-conditional propensity scores. Pharmacoepidemiol Drug Saf.

[49] Luijken K, Spekreijse JJ, van Smeden M (2021). New-user and prevalent-user designs and the definition of study time origin in pharmacoepidemiology: A review of reporting practices. Pharmacoepidemiol Drug Saf.

[50] Hernán MA, Alonso A, Logan R (2008). Observational studies analyzed like randomized experiments: an application to postmenopausal hormone therapy and coronary heart disease. Epidemiology.

[51] Grodstein F, Stampfer MJ, Manson JE (1996). Postmenopausal Estrogen and Progestin Use and the Risk of Cardiovascular Disease. N Engl J Med.

[52] Manson JE, Hsia J, Johnson KC (2003). Estrogen plus Progestin and the Risk of Coronary Heart Disease. N Engl J Med.

[53] Caniglia EC, Zash R, Fennell C (2023). Emulating Target Trials to Avoid Immortal Time Bias - An Application to Antibiotic Initiation and Preterm Delivery. Epidemiology.

[54] Fu EL, Harhay MO, Schneeweiss S (2026). Starting right: aligning eligibility and treatment assignment at time zero when emulating a target trial. BMJ.

[55] Hernán MA, Robins JM (2017). Per-Protocol Analyses of Pragmatic Trials. N Engl J Med.

[56] Boyne DJ, Cheung WY, Hilsden RJ (2021). Association of a Shortened Duration of Adjuvant Chemotherapy With Overall Survival Among Individuals With Stage III Colon Cancer. *JAMA Netw Open*.

[57] Grothey A, Sobrero AF, Shields AF (2018). Duration of Adjuvant Chemotherapy for Stage III Colon Cancer. N Engl J Med.

[58] Wen L, Young JG, Robins JM (2021). Parametric g‐formula implementations for causal survival analyses. Biometrics.

[59] Wanis KN, Sarvet AL, Wen L (2024). Grace periods in comparative effectiveness studies of sustained treatments. J R Stat Soc Ser A Stat Soc.

[60] Khurana V, Bejjanki HR, Caldito G (2007). Statins reduce the risk of lung cancer in humans: a large case-control study of US veterans. Chest.

[61] Dale KM, Coleman CI, Henyan NN (2006). Statins and cancer risk: a meta-analysis. JAMA.

[62] Emberson JR, Kearney PM, Cholesterol Treatment Trialists’ (CTT) Collaboration (2012). Lack of effect of lowering LDL cholesterol on cancer: meta-analysis of individual data from 175,000 people in 27 randomised trials of statin therapy. PLoS ONE.

[63] Danaei G, Rodríguez LAG, Cantero OF (2011). Observational data for comparative effectiveness research: an emulation of randomised trials to estimate the effect of statins on primary prevention of coronary heart disease. Stat Methods Med Res.

[64] Hernán MA, Robins JM (2025). Causal inference: what if?.

[65] Austin PC, Stuart EA (2015). Moving towards best practice when using inverse probability of treatment weighting (IPTW) using the propensity score to estimate causal treatment effects in observational studies. Stat Med.

[66] Kurth T, Walker AM, Glynn RJ (2006). Results of multivariable logistic regression, propensity matching, propensity adjustment, and propensity-based weighting under conditions of nonuniform effect. Am J Epidemiol.

[67] Luque‐Fernandez MA, Schomaker M, Rachet B (2018). Targeted maximum likelihood estimation for a binary treatment: A tutorial. Stat Med.

[68] Bang H, Robins JM (2005). Doubly robust estimation in missing data and causal inference models. Biometrics.

[69] Stuart EA (2019). The reviewer’s guide to quantitative methods in the social sciences.

[70] Stuart EA (2010). Matching Methods for Causal Inference: A Review and a Look Forward. Statist Sci.

[71] Austin PC (2011). An Introduction to Propensity Score Methods for Reducing the Effects of Confounding in Observational Studies. Multivariate Behav Res.

[72] Robins JM, Hernán MÁ, Brumback B (2000). Marginal Structural Models and Causal Inference in Epidemiology. Epidemiology.

[73] Mansournia MA, Etminan M, Danaei G (2017). Handling time varying confounding in observational research. BMJ.

[74] Lund JL, Richardson DB, Stürmer T (2015). The active comparator, new user study design in pharmacoepidemiology: historical foundations and contemporary application. Curr Epidemiol Rep.

[75] Hansford HJ, Cashin AG, Jones MD (2023). Development of the TrAnsparent ReportinG of observational studies Emulating a Target trial (TARGET) guideline. BMJ Open.

[76] Cashin AG, Hansford HJ, Hernán MA (2025). Transparent Reporting of Observational Studies Emulating a Target Trial—The TARGET Statement. JAMA.

[77] Schneeweiss S, Rassen JA, Brown JS (2019). Graphical Depiction of Longitudinal Study Designs in Health Care Databases. Ann Intern Med.

