# Supplementary material for: Identifying and avoiding design related biases in observational studies using the target trial framework
Source: BMJ Med. 2026 Feb 20;5(1):e001280. doi: 10.1136/bmjmed-2024-001280 (PMC12927355; doi:10.1136/bmjmed-2024-001280)
Supplement: Supplementary file 1 [file bmjmed-2024-001280.reviewer_comments.pdf]

PEER REVIEW HISTORY

BMJ Medicine publishes all reviews undertaken for accepted manuscripts. Reviewers are asked to complete a checklist review form and are provided with free text boxes to elaborate on their assessment. These free text comments are reproduced below.

ARTICLE DETAILS

**TITLE (PROVISIONAL)**	Identifying and avoiding design-related biases in observational studies using the target trial framework
**AUTHORS**	Hansford, Harrison; Islam, Nazrul; Lee, Hopin; Dickerman, Barbra; Cashin, Aidan

VERSION 1 - REVIEW

**REVIEWER NAME**	Perol, David
**REVIEWER AFFILIATION**	Centre Leon Berard, Clinical Research
**REVIEWER CONFLICT OF INTEREST**	None
**AI DISCLOSURE**	No
**DATE REVIEW RETURNED**	14-Aug-2025

**GENERAL COMMENTS**	General comments<br>I would like to thank the authors of this manuscript for their efforts in reviewing, clarifying and explaining the main design-related biases in observational studies using the target trial emulation (TTE) framework, and the methodologies used to avoid them.<br><br>This manuscript is of definite educational interest and has many strengths:<br>- Firstly, it is important to clarify the general concepts surrounding these design-related biases, which are often overlooked by clinicians who tend to reduce the issue to that of confounding biases. This is particularly important given that many publications use or attempt to use target trial emulation to evaluate the effectiveness (or real-world efficacy) of therapeutic interventions. However, this is often not true emulation, as there is no systematic matching of the characteristics of the hypothetical randomised trial with the observational database, nor is there sufficient consideration of selection or misclassification biases.<br>- - Secondly, the manuscript itself has many strengths:<br>o The text clearly demonstrates an educational effort by combining a simple yet accurate presentation of complex concepts with useful diagrams to help readers understand. In short, it is a good example of successful synthesis and popularisation.<br>o The bibliography is comprehensive and provides readers with major articles for further reading, including recent works.<br>o - The editorial choices made by the authors are consistent (for example, they do not emphasise the control of confounding factors, whether measured or not, nor do they detail model-based methods such as g-computation).<br><br>However, while reading the manuscript, I was struck by its lack of originality. Many articles on these methods have been published, as well as glossaries that are quite similar to the one provided by the authors in Box 1 (‘key terms’) (see, for example, Rivera DR et al.

JAMA Network Open. 2025;8(6):e2516674). Hernán himself recently published an excellent article (among others!) to further clarify the concepts of target trial emulation (Ann Intern Med. 2025;178:402-407). So, do we really need articles on bias theory in observational studies using the TTE framework and concepts which are now well formalised and widely accepted? Wouldn't we rather see original articles using this methodology to address situations where a randomised controlled trial is not feasible for evaluating treatment effectiveness? In short: enough theory — time for more clinical studies!

Finally, given this article's editorial and educational qualities, I believe it will be very useful to the scientific community — particularly clinicians — in helping them to better understand what I believe to be at the heart of target trial emulation, namely design-related biases. Understanding these concepts is important, and this manuscript will certainly contribute to that.

I would now like to make a few specific comments:
Background:
- I think that the first part of this section (L39 – 51) could be expanded to detail atypical situations in which target trial emulation would be useful. If randomised controlled trials are unavailable, it is often because they are not feasible, either due to ethical considerations or because they involve rare conditions where trials cannot be conducted within a reasonable timeframe. Trials may also be unavailable for the wrong reasons, such as when industrial (and unfortunately, academic) sponsors use poor surrogate endpoints as intermediate criteria, which are unfortunately validated by the FDA and other authorities. As a result, the trial will not be conclusive with regard to the final endpoint. These elements could be explained in more detail or the situations in which trial emulation is clinically useful could be clarified in the introduction.
- The authors rightly state: “However, design-related biases can explain discrepancies between findings in observational data and randomised trials, even more so than confounding” (L 54). This is an important point that is often overlooked by clinicians. Perhaps the authors should cite references from studies that demonstrate this outside the cardiovascular sphere, to show that it is a general consideration. For example, see in oncology Antoine et al., JNCI 2023 and Eur J Cancer 2024.

Identifying common design-related biases in observational studies:
- I think the explanation of the ‘prevalent user bias’ example in the paragraph of the same name could be more detailed, specifically in the last two sentences (L107–113), in order to better inform the reader.
- Similarly, the captions for Figures 2 and 3 (immortal time bias) could be more detailed and explicit.

Avoiding design-related biases with target trial emulation:
- L192 – 194: I fully agree with the authors' view on the practical difficulties of using these emerging designs.
- Well done for explaining the clone-censor-weight approach. Perhaps the authors could provide a clearer definition of a ‘grace period’ and explain its purpose with one or two additional examples.
- As above, the caption for Figure 4 needs to be clarified (for example, the red arrows indicate patients who are no longer compatible with their assigned strategy at baseline…)
- Emulating sequential trials, L243-253: I am sceptical about the

	relevance of this paragraph, because this type of approach actually leads to distorted causal questions of no clinical interest!<br><br>Discussion and Conclusion: I appreciated the paragraph reiterating the need to address confounding or information biases. As in the introduction, however, it would have been helpful if these paragraphs had provided examples of clinical situations in which the TTE approach is particularly useful.

**REVIEWER NAME**	Sharifan, Amin
**REVIEWER AFFILIATION**	University for Continuing Education Krems, Department for Evidence-based Medicine and Evaluation
**REVIEWER CONFLICT OF INTEREST**	None
**AI DISCLOSURE**	No
**DATE REVIEW RETURNED**	16-Aug-2025

**GENERAL COMMENTS**	Thank you for presenting your manuscripts, which aimed to discuss the nuts and bolts of target trial emulation research design. The methodological concepts addressed in your manuscript are not new and have been extensively covered recently in the literature (10.1056/NEJMp2407586; 10.1016/j.jclinepi.2023.10.008; 10.7326/ANNALS-24-01871; 10.1038/s41581-024-00884-4; 10.1001/jama.2022.21383). Therefore, the added value of yet another methods paper that theoretically discusses the concepts of target trial emulation with several examples from the literature is unclear.<br>A major problem I have observed in observational studies is the lack of adherence to the fundamentals of hypothesis testing, which is most commonly used in such studies. This often results in an uninformative and discouraging dichotomization of findings into statistically significant or not. This gap could potentially be addressed by upcoming methods on target trial emulation. Notably, Donald Berry articulated this issue early on in this paper: 10.1038/nrd1927. Likewise, the late Martin Gardner and Douglas Altman elaborated on it decades ago: 10.1136/bmj.292.6522.746. Despite this, these problems persist in the medical literature and warrant resolution, especially as observational studies often lack sample size calculation, identification of primary outcomes, and adjustment for confounding variables.<br>As a side note relevant to your manuscript, in lines 32-33, you equate a study design (presumably the reporting guideline) with critical appraisal. It is important to highlight the major distinctions between assessing a study for bias and ensuring clarity in reporting. A reporting guideline merely facilitates transparency and clarity in the report, whereas assessing the risk of bias (note that the term appraisal is discouraged; see 10.11124/JBIES-22-00434) addresses the methodological quality of a study from various perspectives, an example being ROBINS-I 2 for target trial emulation studies (https://www.riskofbias.info/welcome/robins-i-v2).

**REVIEWER NAME**	Carmine, Thomas
**REVIEWER AFFILIATION**	Praxis Dr. Carmine (Ringgold ID 645326, new), General Medicine
**REVIEWER CONFLICT OF INTEREST**	None

**AI DISCLOSURE**	OpenAI's (ChatGPT/Google) was used to help draft and refine my review's wording. The tool improved clarity, tone, and structure, but all key points, judgments, and recommendations are my own.
**DATE REVIEW RETURNED**	24-Aug-2025

**GENERAL COMMENTS**	Thank you for the opportunity to review this manuscript. This is an important and well-written paper that addresses a critical and often-overlooked area of observational research. The use of clear examples and figures is a major strength. The following comments are intended to help the authors further strengthen the manuscript's clarity, accuracy, and pedagogical impact.<br><br>Overall Comments<br>The manuscript's core strength is its clear focus on design-related biases that arise from the misalignment of time-zero. However, its effectiveness is somewhat limited by a structural issue in the explanation of immortal time, and it misses opportunities to discuss the full methodological complexity of the advanced techniques it advocates. My recommendations below focus on restructuring the central argument on immortal time and adding nuance to the discussion of the proposed methods.<br><br>Major Recommendations<br><br>1. Revise and Restructure the Explanation of 'Immortal Time' for Clarity and Accuracy<br>The manuscript's central argument about immortal time is at times confusing due to a structural issue. A clear, mechanistic explanation is provided on lines 197–202, but it arrives only after the reader has contended with a less-suited abstract definition (lines 116–117) and a potentially confusing first example (Figure 2). A subtle imprecision in phrasing is also repeated throughout the text.<br><br>The Core Issue: The paper repeatedly uses phrases such as "eligibility criteria defined after treatment assignment" (e.g., lines 197–198, 79–80; Box 1). This can be misleading. The bias arises when a criterion's status is ascertained using information that emerges after follow-up begins, regardless of when that criterion was formally defined.<br><br>The Disconnect: The initial definition of immortal time (inability to experience the outcome) does not intuitively fit the first example (Figure 2), where the 'immortality' is with respect to study dropout. This disconnect is only resolved by the later mechanistic explanation.<br><br>Recommendation: Consider restructuring this section so that a corrected and more precise version of the mechanistic explanation (currently lines 197–202) is moved to the beginning of the discussion on immortal time (replacing lines 116–118). This new, operational definition should clearly distinguish between the definition and ascertainment of criteria. Making this change early would resolve inconsistencies and provide a clearer framework that makes both subsequent examples (Figures 2 and 3) and the definitions in Box 1 more immediately understandable.<br><br>2. Address the Full Methodological Complexity of the Proposed Advanced Methods<br>The sections on "Emulating sequential trials" and the "Discussion"

	present only part of the methodological picture, which may leave readers with an incomplete understanding of how these approaches should be applied.<br><br>Time-Varying Confounding: The "Emulating sequential trials" section explains how the method solves immortal time bias through data structure, but it does not mention the essential next step: adjusting for time-varying confounding. Without this, the method remains biased. It would strengthen the section to note that structuring the data is necessary but not sufficient, and that appropriate statistical adjustment (e.g., inverse probability weighting) must also be applied.<br><br>Practical Limitations: The "Discussion" covers general limitations of observational research but does not yet address the specific challenges of the advanced methods presented. A more balanced account could briefly acknowledge pitfalls of the clone-censor-weight approach (e.g., model specification, extreme weights, computational burden) and sequential trial emulation (e.g., complexity of variance estimation).<br><br>3. Refine Key Examples for Methodological Focus and Nuance<br>The illustrative examples are a strength of the manuscript but could be fine-tuned for clarity and focus.<br><br>Prevalent User Bias Example (HRT): This section could benefit from a clearer narrative explaining how early adverse events filter out susceptible patients, leaving a healthier survivor cohort. At present, the conclusion risks oversimplifying the scientific history of HRT and makes a broad statement about the "harm of hormone therapy." A more precise framing would emphasize that, in the specific context of the study cited, correcting for prevalent user bias reversed the study's findings, without making a generalized clinical claim.<br><br>Immortal Time Example (TKR, Figure 2): When restructuring the immortal time section, it would help readers if the authors explicitly acknowledged that this example also contains other biases (e.g., confounding by indication, asymmetrical follow-up). Doing so would help isolate the intended methodological lesson.<br><br>Minor Recommendations<br><br>Title: The current title is accurate, but a more specific version, such as "Using the Target Trial Framework to Avoid Time-Zero Biases in Observational Studies," might better reflect the paper's scope.<br><br>Abstract: The abstract summarizes the paper well but could mention the advanced methods (clone-censor-weight, sequential trials) to reflect the full content.<br><br>Box 1 (Key Terms): The definitions for "clone-censor-weight" and "sequential trial emulation" could be simplified into shorter, more intuitive descriptions to provide a clearer foundation for readers.<br><br>Clone-Censor-Weight Section: The text on lines 234–236 states that censoring occurs "at baseline," but Figure 4 correctly shows it occurs when a patient deviates from their assigned strategy. Revising this would improve alignment.<br><br>Typo: A minor typo exists on line 150 ("before of surgery").

	Overall Assessment<br>This is a valuable manuscript that addresses an important topic with clarity. By restructuring the explanation of immortal time and adding nuance regarding the complexities of advanced methods, the authors could significantly enhance its impact and educational value.

**REVIEWER NAME**	Wang, Bingshun
**REVIEWER AFFILIATION**	Shanghai Jiao Tong University School of Medicine, Department of Biostatistics, Clinical Research Institute, School of Public Health
**REVIEWER CONFLICT OF INTEREST**	None
**AI DISCLOSURE**	In the final stages of preparing this review, the reviewer employed DeepSeek, an AI-assisted tool hosted at Shanghai Jiao Tong University, to refine the readability and clarity of the suggestions. Subsequently, the author thoroughly reviewed and edited the content as necessary, ultimately taking full responsibility for the final version of the review.
**DATE REVIEW RETURNED**	01-Sep-2025

**GENERAL COMMENTS**	General Assessment:<br>The manuscript provides a well-defined explanation of "target trial" and "target trial emulation" (TTE) in observational studies as ways to mitigate design-related biases and enhance causal inference, particularly in contexts where randomized trials are unavailable or impractical. This topic is highly relevant and timely, given the growing reliance on observational data for clinical and policy decisions. From a statistical perspective, TTE in this manuscript does not introduce significant innovative methodological insights, as many of its components have been previously described in the literature. Nevertheless, TTE is valuable as a general framework rather than a specific data analysis method, and it merits broad dissemination to the audience of BMJ Medicine. The paper aligns well with the journal's scope; however, several aspects require improvement to enhance its clarity and impact.<br><br>Specific Recommendations:<br>1. Emphasize Transparency and Reliability<br>• The TTE framework is indeed helpful in making causal questions and the components of the target trial explicit, thereby enhancing the validity of observational study findings. This aligns well with recent efforts to improve transparency in observational research. However, the manuscript should place greater emphasis on how TTE contributes to ensuring the transparency, reproducibility, and reliability of research. Explicitly highlighting these aspects would strengthen the manuscript's alignment with the goals of promoting rigorous and trustworthy research.<br><br>2. Expand on Prospective Data Collection and Real-World Data Quality<br>• The manuscript currently focuses on applying TTE to existing observational datasets. While this is a valid approach, the authors should consider expanding the discussion to explore how TTE can inform the prospective collection of observational data. For instance, how might TTE guide the design of data collection protocols to minimize biases from the outset? Additionally, the manuscript under-emphasizes the quality issues of real-world data, which are critical

	when applying TTE. The authors should address potential limitations and pitfalls associated with using real-world data in TTE, such as missing data, measurement errors, and heterogeneity across datasets. Providing a more nuanced discussion of these challenges would enhance the manuscript's practical relevance.<br><br>3. Provide Real-World Examples of Bias Resolution<br>• The manuscript provides an overview of common design-related biases in TTE, such as selection bias and immortal time bias. While this serves as a useful introduction, the authors could strengthen their argument by incorporating real-world examples that illustrate how TTE effectively addresses these biases in observational studies. For instance, the manuscript already includes a compelling example of resolving "prevalent user bias" in hormone replacement therapy studies, which demonstrates the practical application of TTE. Similarly, the inclusion of concrete case studies that showcase how TTE resolves "immortal time bias" would further enhance the manuscript's credibility and appeal. By grounding theoretical discussions in tangible examples, the authors can more effectively communicate the practical value of TTE and engage readers in a more impactful manner.<br><br>Summary and Final Thoughts:<br>Overall, the manuscript provides a clear and well-defined explanation of the "target trial" and "target trial emulation" concepts, along with a thorough articulation of common design-related biases. This foundational content is valuable for readers unfamiliar with these topics. However, to meet the standards of BMJ Medicine as a "Methods Primer," the manuscript requires refinement in several key areas:<br>• Enhancing transparency and reliability: Explicitly highlight how TTE supports transparent and reproducible research practices.<br>• Expanding on prospective data collection: Discuss how TTE can inform the design of observational studies from the outset.<br>• Addressing real-world data quality issues: Explore the limitations and challenges of applying TTE to real-world datasets.<br>• Providing real-world examples: Illustrate the practical application of TTE through case studies that demonstrate bias resolution.<br><br>By addressing these recommendations, the manuscript will significantly improve its clarity, relevance, and impact, making it a stronger contribution to the field of observational research methodology.<br>____<br>Final Recommendation:<br>The manuscript is suitable for publication after revision. The authors could carefully incorporate the above suggestions to enhance the manuscript's depth, practicality, and alignment with BMJ Medicine's standards. Once revised, the manuscript has the potential to serve as a valuable resource for researchers and practitioners interested in improving the rigor and transparency of observational studies.

**REVIEWER NAME**	Riley, Richard
**REVIEWER AFFILIATION**	University of Birmingham, Institute of Applied Health Research
**REVIEWER CONFLICT OF INTEREST**	None
**AI DISCLOSURE**	No
**DATE REVIEW RETURNED**	18-Sep-2025

GENERAL COMMENTS	Thank you for the opportunity to review this article. I really enjoyed reading it. I am not an expert in casual inference methods or emulated target trial design, so I came at this from a more general background and learnt a lot. It is very well written and clear, with a good flow and I think the BMJ Medicine reader will benefit from it as a resource. I only have a minor suggestion, which is to add examples (or evidence) where the target trial design done well leads to different results from observational studies done badly. That is, to showcase more in real examples how implementing the good practice outlined can actually change findings (and ultimately align more to evidence from RCTs); there must be examples of that type. Currently the examples and figures are about the design aspects (which work well), but if the text had a few real applications and results showing the impact of good practice (in just a few sentences each time), this would greatly benefit the paper.<br><br>Lastly, given the length, I think this paper is more of an traditional Research Methods and Reporting article, than a primer per se

VERSION 1 – AUTHOR RESPONSE

REVIEWER #1:

1. **Background: I think that the first part of this section (L39 – 51) could be expanded to detail atypical situations in which target trial emulation would be useful. If randomised controlled trials are unavailable, it is often because they are not feasible, either due to ethical considerations or because they involve rare conditions where trials cannot be conducted within a reasonable timeframe. Trials may also be unavailable for the wrong reasons, such as when industrial (and unfortunately, academic) sponsors use poor surrogate endpoints as intermediate criteria, which are unfortunately validated by the FDA and other authorities. As a result, the trial will not be conclusive with regard to the final endpoint. These elements could be explained in more detail or the situations in which trial emulation is clinically useful could be clarified in the introduction.**

RESPONSE: We have added citations in our background to highlight the examples of the situations where target trial emulation is clinically useful.

Lines 46 to 50: "The randomised trial is the preferred approach to estimate the causal effects of medical interventions.[1,2] However, it is often not feasible to conduct randomised trials to investigate the long-term safety and effectiveness of interventions,[3] identify specific groups who benefit (or are harmed) most from interventions,[4] or to provide evidence when timely information is needed for decision making.[5]"

2. **The authors rightly state: "However, design-related biases can explain discrepancies between findings in observational data and randomised trials, even more so than confounding" (L 54). This is an important point that is often overlooked by clinicians. Perhaps the authors should cite references from studies that demonstrate this outside the cardiovascular sphere, to show that it is a general consideration. For example, see in oncology Antoine et al., JNCI 2023 and Eur J Cancer 2024.**

RESPONSE: We have added citations of empirical evidence of design related biases to include two studies in oncology (Dickerman et al., Nat Med, 2019; Kuehne et al., JCE 2022).

Lines 63 to 64: "However, design-related biases[12] can explain discrepancies between findings in observational data and randomised trials, even more so than confounding.[13–17]"

3. **Identifying common design-related biases in observational studies: - I think the explanation of the 'prevalent user bias' example in the paragraph of the same name could be more detailed, specifically in the last two sentences (L107–113), in order to better inform the reader.**

RESPONSE: We have added more detail to the explanation of prevalent user bias.

Lines 98 to 104: "Studies that include prevalent users of treatment are prone to selection bias.[37,39] This bias arises when the 'treated' group includes individuals who had been taking treatment for some time before the start of follow-up and who remained alive and event-free until the start of follow-up (Figure 1). This results in a systematic 'depletion of susceptibles' in the 'treated' group, which may therefore appear safer or more effective.

4. **Similarly, the captions for Figures 2 and 3 (immortal time bias) could be more detailed and explicit.**

RESPONSE: We have made the captions of Figure 2 and 3 more detailed and explicit.

Figure 2: "Immortal time due to selection bias. This can arise when eligibility criteria (e.g., 12-month follow-up response) are applied after treatment assignment (e.g., unilateral vs. bilateral total knee replacement [TKR]) and the start of follow-up, thereby creating a period of immortal time between baseline and eligibility ascertainment where the outcome cannot occur for included individuals. Whilst not the focus, this example also includes bias due to misclassification of treatment assignment (see errors in classifying individuals into treatment strategies).

Figure 3: "Immortal time due to misclassification of treatment assignment. This can arise when treatment assignment (e.g., surgery within 6 months or no surgery) occurs after the start of follow up (e.g., cancer diagnosis), which can result in events being misattributed to one of the treatment groups (e.g., above, early deaths marked by blue arrows are misattributed to the no surgery strategy)."

5. **Avoiding design-related biases with target trial emulation: Well done for explaining the clone-censor-weight approach. Perhaps the authors could provide a clearer definition of a 'grace period' and explain its purpose with one or two additional examples.**

RESPONSE: We have added a clearer definition of the grace period and its purpose, highlighting the grace period in the example provided.

Lines 256 to 262: "An example of where the clone-censor-weight approach may be helpful is when comparing treatment strategies that involve a grace period for initiating treatment (that is, a predefined

time after baseline when treatment can begin and the individual would be considered adherent to the treatment strategy throughout this period, reflecting delays common in clinical practice) (Figure 4). Consider the example of comparing receipt of surgery within 6 months of baseline (the grace period) with no surgery over follow-up, among individuals with lung cancer (Figure 3)."

6. **As above, the caption for Figure 4 needs to be clarified (for example, the red arrows indicate patients who are no longer compatible with their assigned strategy at baseline…)**

   RESPONSE: We have clarified this in the caption of Figure 4

   Figure 4: "Illustration of clone-censor-weight approach. Red arrows indicate deviation from treatment strategies leading to censoring (e.g., when individuals classified to the no surgery strategy receive surgery, or when individuals classified to the surgery within a certain grace period strategy do not receive surgery by the end of the grace period)."

7. **Emulating sequential trials, L243-253: I am sceptical about the relevance of this paragraph, because this type of approach actually leads to distorted causal questions of no clinical interest!**

   RESPONSE: We have highlighted the trade-off between avoiding introducing bias and asking a different clinical question in the paragraph (see first set of text, no changes were made). We highlight examples where this approach has been used to avoid introducing bias.

   Lines 296 to 299: "This avoids the introduction of immortal time due to treatment strategy misclassification; however, whether the new causal question (e.g., without grace periods, which may be realistic in many real-world treatment strategies) is still of interest should be considered."

   Lines 312 to 324: "Applying target trial emulation with sequential trial emulation prevented design-related biases in an evaluation of statins and risk of cancer.[56] In earlier observational analyses, investigators reported a substantially lower risk of cancer among statin users compared with non-users (odds ratio for lung cancer of 0.23 (95% CI 0.20 to 0.26), comparing long-term (>4 years) statin users with non-users).[57] These analyses had two flaws previously discussed:[29,50] post-baseline information on achieved duration of statin use was used to classify individuals into treatment strategies, generating immortal time, and prevalent users of treatment were included, leading to selection bias. Dickerman et al. reframed this analysis as a target trial with sequential trial emulation and classified individuals into one of two strategies (statin initiators vs. non-initiators) based on information available at the baseline of each sequential target trial, finding a null effect (hazard ratio = 1.02 [95% CI 0.99 to 1.05]), consistent with meta-analyses of randomized trials.[58,59]

8. **Discussion and Conclusion: I appreciated the paragraph reiterating the need to address confounding or information biases. As in the introduction, however, it would have been helpful if these paragraphs had provided examples of clinical situations in which the TTE approach is particularly useful.**

RESPONSE: We have provided these examples in the introduction, and, since this article is not to highlight the clinical utility of the target trial framework but highlight how it avoids bias we have not added further examples in the discussion.

Lines 46 to 50: "The randomised trial is the preferred approach to estimate the causal effects of medical interventions.[1,2] However, it is often not feasible to conduct randomised trials to investigate the long-term safety and effectiveness of interventions,[3] identify specific groups who benefit (or harmed) most from interventions,[4] or to provide evidence when timely information is needed for decision making.[5]"

REVIEWER #2:

1. **Thank you for presenting your manuscripts, which aimed to discuss the nuts and bolts of target trial emulation research design. The methodological concepts addressed in your manuscript are not new and have been extensively covered recently in the literature (10.1056/NEJMp2407586; 10.1016/j.jclinepi.2023.10.008; 10.7326/ANNALS-24-01871; 10.1038/s41581-024-00884-4; 10.1001/jama.2022.21383). Therefore, the added value of yet another methods paper that theoretically discusses the concepts of target trial emulation with several examples from the literature is unclear.**

   RESPONSE: We have added more detail with a brief outline of the added value of this manuscript.

   Lines 79 to 83: "This Research, Methods and Reporting article aims to provide a detailed outline of how design-related biases occur and how target trial emulation can help avoid these biases, with reference to the growing literature on this topic in health and medical research.[12,13,17,21–27,27–33]. Key terms used in this article are defined in Box 1, with references to articles with more detail.

2. **A major problem I have observed in observational studies is the lack of adherence to the fundamentals of hypothesis testing, which is most commonly used in such studies. This often results in an uninformative and discouraging dichotomization of findings into statistically significant or not. This gap could potentially be addressed by upcoming methods on target trial emulation. Notably, Donald Berry articulated this issue early on in this paper: 10.1038/nrd1927. Likewise, the late Martin Gardner and Douglas Altman elaborated on it decades ago: 10.1136/bmj.292.6522.746. Despite this, these problems persist in the medical literature and warrant resolution, especially as observational studies often lack sample size calculation, identification of primary outcomes, and adjustment for confounding variables.**

   RESPONSE: These issues, whilst critical to interpret observational analyses, go beyond the scope of this paper and are not unique to target trial emulations. To maintain focus and stay within word constraints we haven't elaborated on this in this manuscript.

3. **As a side note relevant to your manuscript, in lines 32-33, you equate a study design (presumably the reporting guideline) with critical appraisal. It is important to highlight the major distinctions between assessing a study for bias and ensuring clarity in reporting. A reporting guideline merely facilitates transparency and clarity in the report, whereas assessing the risk of bias (note that the term appraisal is discouraged; see 10.11124/JBIES-22-00434) addresses the methodological quality of a study from various perspectives, an example being ROBINS-I 2 for target trial emulation studies (https://www.riskofbias.info/welcome/robins-i-v2).**

   RESPONSE: Thank you for this note, we have used the term 'study design' in the abstract, and throughout the text, to refer to how the study was designed and conducted, rather than how it was reported. We have added more text in our discussion to explain how the TARGET guideline is a reporting framework to avoid common confusion between the design of the study, and the reporting of the study.

   Line 358 to 365: "Transparent reporting of target trial emulations is essential to allow readers to appraise, interpret and implement findings in their decision-making. The TARGET guideline [70–72] aims to assist investigators to report relevant information, and support readers to identify key aspects of the target trial emulation. <u>TARGET was not designed to be, and should not be, used as a tool to assess risk of bias or quality of a study nor to guide the conduct of a study. However, although not the intention, the TARGET guideline may help improve study conduct by serving as an educational tool and clarifying key methodologic issues to be addressed by investigators.</u>

REVIEWER #3:

1. **The manuscript's core strength is its clear focus on design-related biases that arise from the misalignment of time-zero. However, its effectiveness is somewhat limited by a structural issue in the explanation of immortal time, and it misses opportunities to discuss the full methodological complexity of the advanced techniques it advocates. My recommendations below focus on restructuring the central argument on immortal time and adding nuance to the discussion of the proposed methods.**

   RESPONSE: We have revised the information provided about immortal time, highlighted in the comments below.

2. **Revise and Restructure the Explanation of 'Immortal Time' for Clarity and Accuracy - The manuscript's central argument about immortal time is at times confusing due to a structural issue. A clear, mechanistic explanation is provided on lines 197–202, but it arrives only after the reader has contended with a less-suited abstract definition (lines 116–117) and a potentially confusing first example (Figure 2). A subtle imprecision in phrasing is also repeated throughout the text.**

   RESPONSE: We have moved the explanation of immortal time and how it can be introduced earlier to the opening paragraph of the section. We have ensured to maintain consistency in phrasing throughout the manuscript.

Lines 107 to 114: “Immortal time refers to a period of time included in the analysis where individuals cannot experience the outcome of interest due to choices about the study design.[12,26,40] In a randomised trial, all outcomes that occur after eligibility ascertainment, treatment strategy assignment, and the start of follow-up are attributed to the appropriate treatment strategy. However, in observational analyses, when information that is ascertained after the start of follow up is used to inform eligibility (or classification into treatment strategies), immortal time may be introduced. Below are examples of specific design decisions that can introduce immortal time.”

3. **The Core Issue: The paper repeatedly uses phrases such as "eligibility criteria defined after treatment assignment" (e.g., lines 197–198, 79–80; Box 1). This can be misleading. The bias arises when a criterion's status is ascertained using information that emerges after follow-up begins, regardless of when that criterion was formally defined.**

   RESPONSE: We have clarified the language around how data that temporally occurs after investigators define the start of follow-up.

   Lines 88 to 95: “Deviation from the design principles of a randomised trial in an observational study will often introduce biases that occur primarily due to the misalignment of ‘time-zero’ when participants initiate treatment, are eligible for inclusion and follow-up begins.[35] For example, selection bias can be introduced when prevalent users of the treatment(s) are included in an analysis,[26] and immortal time may appear when a) eligibility criteria are ascertained with information that emerges after the start of follow-up, or b) information that emerges after baseline is used to classify individuals into a treatment strategy.[20,36,37]

   Box 1: “Errors when classifying individuals into a treatment strategy caused by the use of information that emerges after the beginning of follow-up to assign individuals.”

   Lines 116 to 121: “Using information emerging after treatment assignment to define eligibility (selection bias).

   Using information that emerges after treatment assignment (e.g., adherence to treatment strategy, duration of treatment use, providing follow-up data) to define the eligibility criteria induces selection bias when the analysis starts the follow-up at treatment assignment.”

4. **The Disconnect: The initial definition of immortal time (inability to experience the outcome) does not intuitively fit the first example (Figure 2), where the 'immortality' is with respect to study dropout. This disconnect is only resolved by the later mechanistic explanation.**

   RESPONSE: We have added more information relating to the example and how it illustrates the introduction of immortal time.

Figure 2: “Immortal time due to selection bias. This can arise when eligibility criteria (e.g., 12-month follow-up response) are applied after treatment assignment (e.g., unilateral vs. bilateral total knee replacement [TKR]) and the start of follow-up, thereby creating a period of immortal time between baseline and eligibility ascertainment where the outcome cannot occur for included individuals.”

5. **Recommendation: Consider restructuring this section so that a corrected and more precise version of the mechanistic explanation (currently lines 197–202) is moved to the beginning of the discussion on immortal time (replacing lines 116–118). This new, operational definition should clearly distinguish between the definition and ascertainment of criteria. Making this change early would resolve inconsistencies and provide a clearer framework that makes both subsequent examples (Figures 2 and 3) and the definitions in Box 1 more immediately understandable.**

   RESPONSE: We have revised this section as suggested, as in response #2

   Lines 107 to 114: “Immortal time refers to a period of time included in the analysis where individuals cannot experience the outcome of interest due to choices about the study design.[12,26,40] In a randomised trial, all outcomes that occur after eligibility, treatment assignment and beginning of follow -up are attributed to the appropriate treatment strategy. However, in observational analyses, when information that is ascertained after the start of follow up begins is used to define eligibility (or classification into treatment strategies), immortal time may be introduced. Below are specific design decisions that can introduce immortal time.”

6. **Address the Full Methodological Complexity of the Proposed Advanced Methods. The sections on "Emulating sequential trials" and the "Discussion" present only part of the methodological picture, which may leave readers with an incomplete understanding of how these approaches should be applied.**

   RESPONSE: We believe it is beyond the scope of this paper’s objective and word constraints to provide detailed explanation on the application of these approaches and have instead directed readers to papers that elaborate on how to apply each of these methods, if they wish to do so.

7. **Time-Varying Confounding: The "Emulating sequential trials" section explains how the method solves immortal time bias through data structure, but it does not mention the essential next step: adjusting for time-varying confounding. Without this, the method remains biased. It would strengthen the section to note that structuring the data is necessary but not sufficient, and that appropriate statistical adjustment (e.g., inverse probability weighting) must also be applied.**

   RESPONSE: We have added a statement around the importance time-varying confounding.

Lines 354 to 356: “Similarly, as with all analyses of observational data aiming to estimate causal effects of interventions, time-varying confounding should be addressed using appropriate methods (such as inverse probability weighting[69]) when necessary.”

8. **Practical Limitations: The "Discussion" covers general limitations of observational research but does not yet address the specific challenges of the advanced methods presented. A more balanced account could briefly acknowledge pitfalls of the clone-censor-weight approach (e.g., model specification, extreme weights, computational burden) and sequential trial emulation (e.g., complexity of variance estimation).**

   RESPONSE: We have added more information about the challenges of the proposed methods.

   Lines 338 to 340: “Despite the potential benefits of these approaches, they require rich, longitudinal data and may become computationally intensive in large datasets.”

9. **Refine Key Examples for Methodological Focus and Nuance. The illustrative examples are a strength of the manuscript but could be fine-tuned for clarity and focus.**

   Prevalent User Bias Example (HRT): This section could benefit from a clearer narrative explaining how early adverse events filter out susceptible patients, leaving a healthier survivor cohort. At present, the conclusion risks oversimplifying the scientific history of HRT and makes a broad statement about the "harm of hormone therapy." A more precise framing would emphasize that, in the specific context of the study cited, correcting for prevalent user bias reversed the study’s findings, without making a generalized clinical claim.

   RESPONSE: We have revised this section to more precisely frame the specific context of the study and avoid making a clinical claim.

   Lines 195 to 212: “An example of prevalent-user bias occurred in observational studies investigating hormone replacement therapy for the prevention of coronary heart disease.[50,51] Grodstein et al. (1996) compared women using hormone therapy (prevalent users) to women who did not use hormone therapy. The observational study suggested a reduced risk of coronary heart disease among users of hormone therapy compared to non-users,[51] a result that conflicted with a later randomised trial which showed an increased risk of coronary heart disease for those assigned to initiate hormone therapy compared to placebo.[52] The same data in Grodstein et al. (1996) was used by Hernán et al., (2008) although these investigators adhered more closely to the principles of the randomised trial by comparing initiators to non-initiators and found a similarly increased risk of coronary heart disease to the trial.[50] In the original observational study,[51] the inclusion of prevalent users introduced bias as individuals who were susceptible to adverse events of hormone therapy or who experienced coronary heart disease were less likely to be included in the study (depletion of susceptibles), resulting in an underestimation of the true risk of hormone therapy. The comparison between initiators and non-initiators of treatment better aligns with common clinical decisions about whether to start a new treatment or not, and how a randomised trial would be conducted.[50]

10. Immortal Time Example (TKR, Figure 2): When restructuring the immortal time section, it would help readers if the authors explicitly acknowledged that this example also contains other biases (e.g., confounding by indication, asymmetrical follow-up). Doing so would help isolate the intended methodological lesson.

RESPONSE: We have added a statement that all examples are also subject to confounding, and have made it clear that the example in Figure 2 is also subject to another specific bias.

Lines 93 to 95: "In addition to the design-related biases highlighted in the following sections, other biases such as confounding may also be present and require appropriate data and analytic approaches to address.[38]"

Figure 2: "Immortal time due to selection bias. This can arise when eligibility criteria (e.g., 12-month follow-up response) are applied after treatment assignment (e.g., unilateral vs. bilateral total knee replacement [TKR]) and the start of follow-up, thereby creating a period of immortal time between baseline and eligibility ascertainment where the outcome cannot occur for included individuals. Whilst not the focus, this example also includes bias due to misclassification of treatment assignment (see errors in classifying individuals into treatment strategies).

11. Title: The current title is accurate, but a more specific version, such as "Using the Target Trial Framework to Avoid Time-Zero Biases in Observational Studies," might better reflect the paper's scope.

RESPONSE: Thank you for the suggestion. We have used the term 'design-related biases' to be consistent other literature regarding target trial emulation which separates data-related biases from design-related biases, which are those we outline in the present article. See Hernán et al., Annals of Internal Medicine, 2025

12. Abstract: The abstract summarizes the paper well but could mention the advanced methods (clone-censor-weight, sequential trials) to reflect the full content.

RESPONSE: We decided not to include these approaches in the abstract as they are not the focus of the paper and the abstract aims to provide a broad overview of the key points of the article. We have left technical details to the main text.

13. Box 1 (Key Terms): The definitions for "clone-censor-weight" and "sequential trial emulation" could be simplified into shorter, more intuitive descriptions to provide a clearer foundation for readers.

RESPONSE: Given these terms may be new for readers who are unfamiliar with target trial emulation and these approaches, we believe it is important to provide an outline of the multi-step procedure that

each of these approaches are. We have maintained a length which we believe captures the minimum but essential information required for readers.

14. **Clone-Censor-Weight Section: The text on lines 234–236 states that censoring occurs "at baseline," but Figure 4 correctly shows it occurs when a patient deviates from their assigned strategy. Revising this would improve alignment.**

    RESPONSE: We have added more information to highlight that cloning happens at baseline, but censoring occurs when the patient deviates from their assigned strategy.

    Lines 239 to 246: "An approach to handle questions where treatment strategies are indistinguishable at time-zero, in conjunction with the target trial framework, is the clone-censor-weight approach.[30,32] To implement this, investigators can clone participants, that is, replicate individuals, and assign replicates to as many treatment strategies as their data are compatible with at baseline. As a person's observed treatment pattern becomes incompatible with their assigned strategy, their follow up in that treatment strategy is artificially stopped or 'censored' by the investigator<u> at the time of deviation from their assigned strategy</u>."

15. **Typo: A minor typo exists on line 150 ("before of surgery").**

    RESPONSE: Thank you, we have removed 'of'

    Lines 146 to 148: "Therefore, in this scenario, events (e.g., death) that occur before surgery will be attributed to the no surgery strategy"

REVIEWER #4:

1. **Emphasize Transparency and Reliability: The TTE framework is indeed helpful in making causal questions and the components of the target trial explicit, thereby enhancing the validity of observational study findings. This aligns well with recent efforts to improve transparency in observational research. However, the manuscript should place greater emphasis on how TTE contributes to ensuring the transparency, reproducibility, and reliability of research. Explicitly highlighting these aspects would strengthen the manuscript's alignment with the goals of promoting rigorous and trustworthy research.**

    RESPONSE: We have added more information about the importance of clear reporting of a target trial emulation with the TARGET guideline, and how this supports transparency and may improve study conduct.

    Lines 357 to 366: "Transparent reporting of target trial emulations is essential to allow readers to appraise, interpret and implement findings in their decision-making. The TARGET guideline [69–71] aims to assist investigators to report relevant information, and support readers to identify key aspects

of the target trial emulation. TARGET was not designed to be, and should not be, used as a tool to assess risk of bias or quality of a study. However, although not the intention, the TARGET guideline may help improve study conduct by serving as an educational tool and clarifying key methodologic issues to be addressed by investigators. Study design diagrams, such as those described by Schneeweiss et al., (2019), can also assist investigators to communicate the timing of different aspects of the study design.[73]"

2. **Expand on Prospective Data Collection and Real-World Data Quality - The manuscript currently focuses on applying TTE to existing observational datasets. While this is a valid approach, the authors should consider expanding the discussion to explore how TTE can inform the prospective collection of observational data. For instance, how might TTE guide the design of data collection protocols to minimize biases from the outset? Additionally, the manuscript under-emphasizes the quality issues of real-world data, which are critical when applying TTE. The authors should address potential limitations and pitfalls associated with using real-world data in TTE, such as missing data, measurement errors, and heterogeneity across datasets. Providing a more nuanced discussion of these challenges would enhance the manuscript's practical relevance.**

   RESPONSE: This manuscript aims to address the issue of design-related biases and the value of target trial emulation in avoiding these. Whilst common, it is not part of the framework that dictates whether retrospective or prospective data are used. Issues of data quality are beyond the scope of this article, however, we have briefly stated them so readers are aware of these issues.

   Lines 30 to 34: "Conceptualising the analysis of observational data to estimate the causal effects of interventions as an attempt to explicitly emulate a target trial can help avoid design-related biases, so that investigators can instead and focus on data-related biases (e.g., confounding, measurement error) not directly addressed by the framework."

3. **Provide Real-World Examples of Bias Resolution - The manuscript provides an overview of common design-related biases in TTE, such as selection bias and immortal time bias. While this serves as a useful introduction, the authors could strengthen their argument by incorporating real-world examples that illustrate how TTE effectively addresses these biases in observational studies. For instance, the manuscript already includes a compelling example of resolving "prevalent user bias" in hormone replacement therapy studies, which demonstrates the practical application of TTE. Similarly, the inclusion of concrete case studies that showcase how TTE resolves "immortal time bias" would further enhance the manuscript's credibility and appeal. By grounding theoretical discussions in tangible examples, the authors can more effectively communicate the practical value of TTE and engage readers in a more impactful manner.**

   RESPONSE: We have added an example of empirical evidence on the impact of immortal time bias and how target trial emulation helps avoid it.

   Lines 222 to 229: "Immortal time can have substantial impacts on study results. To illustrate this, Kuehne et al., systematically applied different approaches to estimate the effect of LOT2 (a type of chemotherapy for ovarian cancer), including designs subject to immortal time, to highlight how the estimates change with design decisions.[17] With the 'naïve' approach subject to immortal time, they estimated a hazard ratio of 0.56 (95% CI 0.49 to 0.64). By contrast, when they emulated the design of

an index trial, they estimated a hazard ratio of 1.12 (95% CI 0.96 to 1.28), which was compatible with the results of the trial.[17]"

REVIEWER #5:

1. **I only have a minor suggestion, which is to add examples (or evidence) where the target trial design done well leads to different results from observational studies done badly. That is, to showcase more in real examples how implementing the good practice outlined can actually change findings (and ultimately align more to evidence from RCTs); there must be examples of that type. Currently the examples and figures are about the design aspects (which work well), but if the text had a few real applications and results showing the impact of good practice (in just a few sentences each time), this would greatly benefit the paper.**

   RESPONSE: We have added two empirical examples of where the approaches discussed have lead to importantly different results from earlier, flawed observational analyses.

   Line 272 to 285: "Applying target trial emulation with cloning, censoring and weighting prevented immortal time in a recent example by Boyne et al. investigating survival following short or longer duration of adjuvant chemotherapy for colon cancer.[54] Prior to this study, findings from a randomised trial (the IDEA trial) [54] conflicted with those from observational studies. In these previous observational studies, information emerging after baseline (achieved duration of treatment) was used to classify individuals into treatment strategies, leading to immortal time. Boyne et al. 2021, demonstrated that with the 'naïve' approach taken in previous observational studies, estimates suggested that shorter durations of treatment substantially worsened survival (hazard ratio = 3.33 [95% CI 1.04 to 10.65]).[54] However, when information emerging after baseline was not used to classify individuals into treatment strategies, and instead cloning, censoring and weighting was applied, the estimates were compatible with those from the IDEA trial (emulation hazard ratio = 0.96 [95% CI 0.43 to 2.14], trial hazard ratio = 0.96 [95% CI 0.85 to 1.08]).[54]"

   Lines 311 to 323: "Applying target trial emulation with sequential trial emulation prevented design-related biases in an evaluation of statins and risk of cancer.[56] In earlier observational analyses, investigators reported a substantially lower risk of cancer among statin users compared with non-users (odds ratio for lung cancer of 0.23 (95% CI 0.20 to 0.26), comparing long-term (>4 years) statin users with non-users).[57] These analyses had two flaws previously discussed:[29,50] post-baseline information on achieved duration of statin use was used to classify individuals into treatment strategies, generating immortal time, and prevalent users of treatment were included, leading to selection bias. Dickerman et al. reframed this analysis as a target trial with sequential trial emulation and classified individuals into one of two strategies (statin initiators vs. non-initiators) based on information available at the baseline of each sequential target trial, finding a null effect (hazard ratio = 1.02 [95% CI 0.99 to 1.05]), consistent with meta-analyses of randomized trials.[58,59]"

2. **Lastly, given the length, I think this paper is more of an traditional Research Methods and Reporting article, than a primer per se**

RESPONSE: We agree and were unable to change this in the online portal, but have asked the editorial staff to change the article type from Methods primer to a Research Methods and Reporting article.

VERSION 2 – REVIEW

**REVIEWER NAME**	Perol, David
**REVIEWER AFFILIATION**	Centre Leon Berard, Clinical Research
**REVIEWER CONFLICT OF INTEREST**	None
**AI DISCLOSURE**	No
**DATE REVIEW RETURNED**	19-Nov-2025

**GENERAL COMMENTS**	I would like to thank the authors for taking my comments, and those of the other reviewers, into account. Clarifying the methodological explanations and figures, and adding appropriate examples and references, has improved the manuscript's value.<br>Furthermore, the authors have generally retained the initial structure of their work, which suited their objectives of enlightening novice and experienced readers alike about the biases affected by target trial emulation.<br>I have no further comments, as I believe the manuscript is now ready for publication.

**REVIEWER NAME**	Carmine, Thomas
**REVIEWER AFFILIATION**	Praxis Dr. Carmine (Ringgold ID 645326, new), General Medicine
**REVIEWER CONFLICT OF INTEREST**	None
**AI DISCLOSURE**	A Large Language Model (LLM) was used to structure and draft the review comments based on my specific scientific inputs. The AI assisted in refining the language for clarity and conciseness, while all critical evaluation and final verification remained with the reviewer.
**DATE REVIEW RETURNED**	22-Nov-2025

**GENERAL COMMENTS**	I have reviewed the extensively revised manuscript and the authors' detailed responses. I am pleased to report that the authors have effectively addressed the substantive methodological and structural concerns raised in the previous round of review.<br><br>Specifically, the restructuring of the "Immortal Time" section to lead with a clear mechanistic explanation, the precision regarding the ascertainment of eligibility criteria, and the inclusion of necessary caveats regarding time-varying confounding and computational challenges have significantly strengthened the paper.<br><br>The definitions and examples are now logically consistent and methodologically accurate.<br>I have no further major concerns.<br><br>However, before final publication, I recommend correcting the following minor proofreading errors found in the revised text:

	Line 32: There is an extra word in the phrase. It currently reads "...can instead and focus on..."; it should likely read "...can instead focus on...".<br>Line 111: For consistency with the rest of the manuscript, "follow up" should be hyphenated as a noun ("...start of follow-up...").<br>Line 330: The sentence structure is grammatically incomplete ("...however, is not described in this article..."). It should be revised for flow, for example: "...however, it is not described in this article..."<br><br>With these minor corrections, I believe the manuscript is ready for publication.

**REVIEWER NAME**	Riley, Richard
**REVIEWER AFFILIATION**	University of Birmingham, Institute of Applied Health Research
**REVIEWER CONFLICT OF INTEREST**	None
**AI DISCLOSURE**	No
**DATE REVIEW RETURNED**	07-Dec-2025

**GENERAL COMMENTS**	I really enjoyed reading this RMR paper for BMJ Medicine. It is rare to have such a well written RMR submission, and I applaud the authors for tackling an important subject matter with clear writing, examples, and flow for a broad audience.<br><br>I am not a causal inference expert, so am reviewing this from a more general statistical background, with experience of writing RMR articles. I hope the following comments help the authors to put the final touches to their RMR, to ensure it has the biggest impact.<br><br>1) “design-related biases[12] can explain discrepancies between findings in<br>observational data and randomised trials, even more so than confounding.” – I would say ‘sometimes’ even more than confounding, because as written it sounds like it is always more than confounding, which clearly is not necessarily the case<br><br>2) “It is critical that readers and investigators alike can identify and avoid these often-<br>overlooked design-related biases” – I would add reviewers to this list, maybe even decision or policy makers too. That is, sell the importance to stakeholders more strongly.<br><br>3) In the first introduction paragraph of ‘Identifying common design-related biases in observational studies’ section, the authors start introducing selection bias and immortal time bias very briefly – in regards to misalignment of time zero. However, it feels like these are quickly dropped and left, before then moving onto the ‘main’ items following the sub-headings of ‘prevalent user bias, etc’ – this is very confusing. Why not simply say in the 1st paragraph that this section covers key design related biases, and then include time zero as the first item(s)? Otherwise it is lost for the reader. Maybe the issue is that this first paragraph starts to give examples (e.g. selection bias and immortal time bias) that are then considered later anyway.<br><br>4) A related point, the authors suddenly mention at end of the first paragraph that confounding bias analysis issues are not covered in the article. It is quite hard to disentangle the analysis approach from

	the design, and indeed later some aspects of analysis, like clone censor weights, are still discussed. I would suggest to simply mention at the end of the introduction, where the aims of the article are specified, that you don't cover all analysis issues in this article, but only those related to the design aspect. Or something like that? Also, ideally add a short paragraph before the discussion, signposting readers to good analysis approaches that should be used to adjust for confounding, linked to the design aspects mentioned; this would be more complete, obviously without going into much detail. Reporting guidelines might be mentioned at that point too.<br><br>5) 'Below are examples of specific design decisions that can introduce immortal time' – these are given as underlined headings, but this does not help with the flow of sections and sub-sections for the reader. To help, suggest the main design bias issues are given as numbers (i), (ii) etc to and then these examples of design decisions are given as bullet points.<br><br>6) For each type of bias, we need an example as they are not always given. Eg. In the prevalent user bias section.<br><br>Often a running example throughout the whole study would help with RMR papers, but I think that would be difficult here, given that one study is unlikely to demonstrate all the issues required. Hence, make sure examples are littered throughout when introducing new ideas.<br><br>7) A major comment is that the authors do not always tell us about the (likely) direction of bias, for the various items. Are these biases (mostly) leading to larger treatment effect estimates? Or could they act in both directions? The reader needs to know the impact. With confounding bias, we generally know effect estimates will be inflated (systematically too big), but what about design bias?<br><br>8) In the 'Avoiding design-related biases with target trial emulation' section, there is also an issue with the sub-headings. Some are italics, and then some are underlined. The flow is lost. I am not sure how to address this, but using (i), (ii) etc and also bullet points may help, as above.<br><br>9) Most RMR articles do not end with a 'Discussion' section, and I found this long and distracting. If there are key aspects in the discussion that were not mentioned before, then these should be embedded in earlier sections (see suggestion earlier about a short section on analysis methods, which will help tackle this issue – the reporting discussion could be moved into that part too). Rather, focus on a few 'concluding remarks' sections to complete the article.<br><br>I look forward to reading the revision.

VERSION 2 – AUTHOR RESPONSE

Reviewer 1:

I would like to thank the authors for taking my comments, and those of the other reviewers, into account. Clarifying the methodological explanations and figures, and adding appropriate examples and references, has improved the manuscript's value.

Furthermore, the authors have generally retained the initial structure of their work, which suited their objectives of enlightening novice and experienced readers alike about the biases affected by target trial emulation.

I have no further comments, as I believe the manuscript is now ready for publication.

Response: Thank you.

Reviewer: 3

Line 32: There is an extra word in the phrase. It currently reads "...can instead and focus on..."; it should likely read "...can instead focus on...".

RESPONSE: We have removed "and"

Line 34 to 35: "so that investigators can instead focus on data-related biases (e.g., confounding, measurement error) not directly addressed by the framework."

Line 111: For consistency with the rest of the manuscript, "follow up" should be hyphenated as a noun ("...start of follow-up...").

RESPONSE: We have updated this throughout the manuscript.

Line 330: The sentence structure is grammatically incomplete ("...however, is not described in this article..."). It should be revised for flow, for example: "...however, it is not described in this article..."

RESPONSE: We have updated the sentence as suggested .

Lines 299 to 301: "Aside from the clone-censor-weight approach, we note that the plug-in g-formula is another approach available to analysts; however, it is not described in this article with further reading available elsewhere.[57,58]"

Reviewer: 5

1) "design-related biases[12] can explain discrepancies between findings in observational data and randomised trials, even more so than confounding." – I would say 'sometimes' even more than confounding, because as written it sounds like it is always more than confounding, which clearly is not necessarily the case.

RESPONSE: We agree and have updated the sentence with "sometimes" as suggested.

Lines 73 to 75: "However, design-related biases[12] can explain discrepancies between findings in observational data and randomised trials, <u>sometimes</u> even more so than confounding.[13–17]"

2) "It is critical that readers and investigators alike can identify and avoid these often-overlooked design-related biases" – I would add reviewers to this list, maybe even decision or policy makers too. That is, sell the importance to stakeholders more strongly.

RESPONSE: We have expanded the 'readers' to make explicit some of the stakeholders for whom this is important

Lines 87 to 90: "It is critical that <u>all</u> <u>investigators, and</u> readers, <u>including clinicians, peer reviewers, and other health and policy decision-makers</u>, can identify and avoid these often-overlooked design-related biases to maximise the usefulness of observational data for decision-making.<u>[21,22]</u>"

3) In the first introduction paragraph of 'Identifying common design-related biases in observational studies' section, the authors start introducing selection bias and immortal time bias very briefly – in regards to misalignment of time zero. However, it feels like these are quickly dropped and left, before then moving onto the 'main' items following the sub-headings of 'prevalent user bias, etc' – this is very confusing. Why not simply say in the 1st paragraph that this section covers key design related biases, and then include time zero as the first item(s)? Otherwise it is lost for the reader. Maybe the issue is that this first paragraph starts to give examples (e.g. selection bias and immortal time bias) that are then considered later anyway.

RESPONSE: We have revised the introduction to this section to highlight that all these design-related biases stem from the misalignment of time zero. The concept of which is clarified in the introduction

Lines 71 to 106: "Clinicians and policy makers are often hesitant to rely on evidence from observational studies due to concerns of confounding bias due to the lack of randomisation.[10,11] However, design-related biases[12] can explain discrepancies between findings in observational data and randomised trials, <u>sometimes</u> even more so than confounding.[13–17] These biases are introduced when observational studies are designed in a manner which deviates from the design principles of a randomised trial.[18] For example, well-designed randomised trials will follow participants from the time of randomisation (treatment strategy assignment) immediately after meeting eligibility criteria, <u>such that there is a clear 'time-zero.'</u> In observational studies of longitudinal data, the timing of eligibility, treatment strategy assignment and start of follow-up are not naturally aligned, and analysts must therefore define these timepoints retrospectively.[25] The choice of these timepoints is where errors commonly occur.[13,19] In a cross-sectional study of 200 observational studies of drug

interventions,[20] Yaacoub et al., highlighted that over 75% of studies were subject to at least one avoidable design-related bias caused by the misalignment of time-zero.

It is critical that all investigators, and readers, including clinicians, peer reviewers, and other health and policy decision-makers, can identify and avoid these often-overlooked design-related biases to maximise the usefulness of observational data for decision-making.[21,22]" This article aims to provide a detailed outline of how design-related biases may occur and how target trial emulation can help avoid these biases, with reference to the growing literature on this topic in health and medical research.[12,13,17,24,25,19,26–29,29–35] We note that, while target trial emulation can help to avoid design-related biases, it does not eliminate biases inherent to the observational data, such as confounding.[25] Whilst not the focus of this article, we briefly discuss approaches to handle confounding.[36] We also highlight the importance of transparent reporting for observational studies of interventions to be appraised and used in practice.[37] Key terms used in this article are defined in Box 1, with references to articles with more detail.

Identifying common design-related biases in observational studies

The design-related biases described in this section all stem from deviation from the design principles of a randomised trial. These include selection bias due to the inclusion of prevalent users (Section i) and immortal time generated when information after the start of follow-up is used to ascertain eligibility or classify individuals into treatment strategies (Sections iia, iib)."

4) A related point, the authors suddenly mention at end of the first paragraph that confounding bias analysis issues are not covered in the article. It is quite hard to disentangle the analysis approach from the design, and indeed later some aspects of analysis, like clone censor weights, are still discussed. I would suggest to simply mention at the end of the introduction, where the aims of the article are specified, that you don't cover all analysis issues in this article, but only those related to the design aspect. Or something like that? Also, ideally add a short paragraph before the discussion, signposting readers to good analysis approaches that should be used to adjust for confounding, linked to the design aspects mentioned; this would be more complete, obviously without going into much detail. Reporting guidelines might be mentioned at that point too.

RESPONSE: We have moved the point on confounding to the end of the introduction and added a section before the discussion on approaches to handle confounding and reporting guidelines.

Lines 87 to 99: "It is critical that all investigators, and readers, including clinicians, peer reviewers, and other health and policy decision-makers, can identify and avoid these often-overlooked design-related biases to maximise the usefulness of observational data for decision-making.[21,22]" This article aims to provide a detailed outline of how design-related biases may occur and how target trial emulation can help avoid these biases, with reference to the growing literature on this topic in health and medical research.[12,13,17,24,25,19,26–29,29–35] We note that, while target trial emulation can help to avoid design-related biases, it does not eliminate biases inherent to the observational data, such as confounding.[25] Whilst not the focus of this article, we briefly discuss approaches to handle confounding.[36] We also highlight the importance of transparent reporting for observational studies of interventions to be appraised and used in practice.[37] Key terms used in this article are defined in Box 1, with references to articles with more detail."

Lines 342 to 360: "Common approaches to handle confounding

It is important to note that, while target trial emulation can help to avoid the design-related biases outlined above, it does not eliminate data-related biases, such as confounding or measurement error.[46] Such biases must be addressed through careful measurement and adjustment for key variables via appropriate analytic techniques in combination with unverifiable assumptions.[65] Analytic approaches to adjust for confounding may involve modelling the probability of treatment (e.g., inverse probability of treatment weighting[66]), the probability of the outcome (e.g., multivariable outcome regression,[68] the g-formula),[58,59] or both (e.g., doubly-robust approaches such as targeted maximum likelihood estimation[69] and augmented inverse probability weighting[70]). Matching, often on the propensity score,[71,72] may be used to adjust for confounding of point-interventions but is not appropriate for time-varying settings.[73,74] Confounding by indication may be more of a concern when comparing an active intervention (e.g., a drug) to no intervention; the use of an active comparator (e.g., another drug) may reduce the magnitude of confounding by indication.[75] Similarly, as with all analyses aiming to estimate causal effects of sustained intervention strategies, time-varying confounding should be addressed using appropriate methods (such as inverse probability weighting[74]) when necessary."

Lines 363 to 373: "Observational studies of interventions should be considered attempts to emulate a target trial,[25,43] and the design-related issues described in this manuscript are easier to assess when authors explicitly report their analysis as a target trial emulation.[21] The TARGET guideline [37,76,77] aims to assist investigators to report relevant information, and support readers to identify key aspects of the target trial emulation. TARGET was not designed to be, and should not be, used as a tool to assess risk of bias or quality of a study. However, although not the intention, the TARGET guideline may help improve study conduct by serving as an educational tool and clarifying key methodologic issues to be addressed by investigators. Study design diagrams, such as those described by Schneeweiss et al., (2019), can also assist investigators to communicate the timing of different aspects of the study design.[78]"

5) 'Below are examples of specific design decisions that can introduce immortal time' – these are given as underlined headings, but this does not help with the flow of sections and sub-sections for the reader. To help, suggest the main design bias issues are given as numbers (i), (ii) etc to and then these examples of design decisions are given as bullet points.

RESPONSE: We have added numbering to this section to clarify the structure.

6) For each type of bias, we need an example as they are not always given. Eg. In the prevalent user bias section.

Often a running example throughout the whole study would help with RMR papers, but I think that would be difficult here, given that one study is unlikely to demonstrate all the issues required. Hence, make sure examples are littered throughout when introducing new ideas.

RESPONSE: We have organised the article so when the biases are introduced, the figures outline the structure of how the biases arise, then, in the 'Avoiding design-related biases with target trial

emulation' section, we provide empirical examples on the impact of the biases and the results when they are avoided. An example for prevalent user bias is from Lines 206 to 223.

7) A major comment is that the authors do not always tell us about the (likely) direction of bias, for the various items. Are these biases (mostly) leading to larger treatment effect estimates? Or could they act in both directions? The reader needs to know the impact. With confounding bias, we generally know effect estimates will be inflated (systematically too big), but what about design bias?

RESPONSE: Whilst we agree it would be very helpful to have be able to predict the direction and magnitude of these biases, it is dependent on many factors including the clinical context / question. We have highlighted in the manuscript.

Lines 106 to 110: "Whilst desirable, it is challenging to predict the magnitude and direction of these design-related biases.[38] We hope by providing the structure of how these biases occur in this article, readers may be better placed to assess the potential direction of bias in the context of their own research questions or studies they may appraise."

8) In the 'Avoiding design-related biases with target trial emulation' section, there is also an issue with the sub-headings. Some are italics, and then some are underlined. The flow is lost. I am not sure how to address this, but using (i), (ii) etc and also bullet points may help, as above.

RESPONSE: We have added numbers to better define the structure of the section.

Lines 102 to 106: The design-related biases described in this section all stem from deviation from the design principles of a randomised trial. These include selection bias due to the inclusion of prevalent users (Section i) and immortal time generated when information after the start of follow-up is used to ascertain eligibility or classify individuals into treatment strategies (Sections iia, iib)."

Lines 188 to 192: There are several approaches under the target trial framework to help investigators avoid inducing design-biases common in observational studies, such as to avoid inclusion of individuals already taking treatment at baseline (1. Avoiding prevalent user bias) and to avoid the use of information that is available after baseline to assign individuals to treatment strategies or to define eligibility (2. Avoiding immortal time).

9) Most RMR articles do not end with a 'Discussion' section, and I found this long and distracting. If there are key aspects in the discussion that were not mentioned before, then these should be embedded in earlier sections (see suggestion earlier about a short section on analysis methods, which will help tackle this issue – the reporting discussion could be moved into that part too). Rather, focus on a few 'concluding remarks' sections to complete the

article.

RESPONSE: We have removed the discussion and included important information on confounding and reporting in the main text.

Lines 342 to 382: "Common approaches to handle confounding

It is important to note that, while target trial emulation can help to avoid the design-related biases outlined above, it does not eliminate data-related biases, such as confounding or measurement error.[46] Such biases must be addressed through careful measurement and adjustment for key variables via appropriate analytic techniques in combination with unverifiable assumptions.[65] Analytic approaches to adjust for confounding may involve modelling the probability of treatment (e.g., inverse probability of treatment weighting[66]), the probability of the outcome (e.g., multivariable outcome regression,[68] the g-formula),[58,59] or both (e.g., doubly-robust approaches such as targeted maximum likelihood estimation[69] and augmented inverse probability weighting[70]). Matching, often on the propensity score,[71,72] may be used to adjust for confounding of point-interventions but is not appropriate for time-varying settings.[73,74] Confounding by indication may be more of a concern when comparing an active intervention (e.g., a drug) to no intervention; the use of an active comparator (e.g., another drug) may reduce the magnitude of confounding by indication.[75] Similarly, as with all analyses aiming to estimate causal effects of sustained intervention strategies, time-varying confounding should be addressed using appropriate methods (such as inverse probability weighting[74]) when necessary."

Transparent reporting of observational studies of interventions

Observational studies of interventions should be considered attempts to emulate a target trial,[25,43] and the design-related issues described in this manuscript are easier to assess when authors explicitly report their analysis as a target trial emulation.[21] The TARGET guideline [37,76,77] aims to assist investigators to report relevant information, and support readers to identify key aspects of the target trial emulation. TARGET was not designed to be, and should not be, used as a tool to assess risk of bias or quality of a study. However, although not the intention, the TARGET guideline may help improve study conduct by serving as an educational tool and clarifying key methodologic issues to be addressed by investigators. Study design diagrams, such as those described by Schneeweiss et al., (2019), can also assist investigators to communicate the timing of different aspects of the study design.[78]"

Conclusion

In settings where trials are unavailable or infeasible, observational studies may provide evidence to address the relevant clinical question. Design-related biases are common in observational studies on the comparative benefits and harms of interventions, but explicit emulation of a target trial can help investigators avoid these biases and enable readers to identify them when present. The use of the target trial framework and transparent reporting of these studies may support sound policy and clinical decisions from observational studies."
